# Natural antisense transcript of natriuretic peptide precursor A (*NPPA*): structural organization and modulation of *NPPA *expression

**DOI:** 10.1186/1471-2199-10-81

**Published:** 2009-08-11

**Authors:** Tarmo Annilo, Katrin Kepp, Maris Laan

**Affiliations:** 1 Institute of Molecular and Cell Biology, University of Tartu, Riia 23, 51010 Tartu, Estonia

## Abstract

**Background:**

Mammalian transcriptome contains a large proportion of diverse and structurally complex noncoding RNAs. One class of such RNAs, natural antisense transcripts (NATs), are derived from the opposite strand of many protein-coding genes. Although the exact structure and functional relevance of most of the NATs is unknown, their emerging role as gene expression regulators raises the hypothesis that NATs might contribute to development of complex human disorders. The goal of our study was to investigate the involvement of NATs in regulation of candidate genes for blood pressure.

**Results:**

First we analysed blood pressure candidate genes for the presence of natural antisense transcripts. *In silico *analysis revealed that seven genes (*ADD3*, *NPPA*, *ATP1A1*, *NPR2*, *CYP17A1*, *ACSM3*, *SLC14A2*) have an antisense partner transcribed from the opposite strand. We characterized *NPPA *and its antisense transcript (*NPPA-AS*) in more detail. We found that *NPPA-AS *is expressed in a number of human tissues as a collection of alternatively spliced isoforms and that *NPPA-AS *and *NPPA *can form RNA duplexes *in vivo*. We also demonstrated that a specific *NPPA-AS *isoform is capable of down-regulating the intron-retained *NPPA *mRNA variant. We studied the evolutionary conservation of *NPPA-AS *and were able to detect the presence of *Nppa-as *transcript in mouse.

**Conclusion:**

Our results demonstrate functional interaction of *NPPA-AS *with *NPPA *at the RNA level and suggest that antisense transcription might be an important post-transcriptional mechanism modulating *NPPA *expression.

## Background

A number of large-scale transcriptional mapping studies have shown that the mammalian transcriptome is extremely complex not only due to alternative splicing but also (and maybe primarily) because of the abundance of noncoding and often overlapping transcriptional units [1-4]. This has raised the hypothesis of RNA-based regulatory system that has allowed the elaboration and expansion of phenotypic complexity of multicellular organisms [5]. It appears that the transcription from both strands in eukaryotic genomes is widespread [6-10], resulting in a large pool of complementary RNAs, or natural sense-antisense transcript pairs. The diversity and extent of antisense transcription suggests that this may be an important and common mechanism of gene expression modulation (recently reviewed in [11-13]).

Depending on the methodological approach and criteria for antisense transcript detection, the estimates of the proportion of transcripts involved in formation of sense-antisense pairs varies from 20 to 40% [2,6-10]. Majority of the natural antisense transcripts (NATs) originate from the opposite DNA strand of the same locus as the sense transcript (cis-NATs). In some cases, NATs can be transcribed from different loci on the genome (trans-NATs) [14]. Although high-throughput studies have investigated expression pattern and evolution of antisense transcripts on a genome-wide scale, the direct regulatory role of NATs has been demonstrated only in a few cases. The mode of NAT action includes very different mechanisms like transcriptional interference [15], RNA masking [16], and epigenetic silencing by triggering heterochromatin formation [17]. In addition, other double-stranded RNA dependant mechanisms like RNA editing or RNA interference may be involved. It has been shown that bidirectionally transcribed loci in mouse can produce endogenous siRNAs [14] and therefore may regulate gene expression by means of RNAi. In the case of *Zeb2 *(zinc finger E-box binding homeobox 2) expression regulation, a NAT masks one of the 5' splice sites of *Zeb2 *pre-mRNA, thereby causing the retention of regulatory intron that is necessary for the translation of Zeb2 protein [16]. Strong phenotypic effect of antisense transcription was shown in a specific case of thalassemia which is caused by a deletion leading to aberrant antisense transcription and silencing of a neighboring gene by CpG island methylation [18]. The potential role of NATs in the regulation of gene expression raises the hypothesis that they might contribute to complex genetic human disorders such as cardiovascular disease, cancer, diabetes or mental disorders.

The goal of the present study was to investigate whether natural antisense transcripts are involved in regulation of candidate genes for hypertension. We proposed that the functional variation of candidate genes might be affected by the interaction with regulatory factors, including non-coding antisense RNAs. We focused on the genes with demonstrated role in familial forms of hypo- and hypertension from a salt-water homeostasis pathway [19-21].

We identified seven genes that are associated with cis-NATs (*ADD3*, *NPPA*, *ATP1A1*, *NPR2*, *CYP17A1*, *ACSM3*, *SLC14A2*). Detailed analysis was carried out for *NPPA *(natriuretic peptide precursor A) and its natural antisense transcript, *NPPA-AS*. *NPPA *codes for a precursor of atrial natriuretic peptide (ANP) that protects the cardiovascular system from the volume and pressure overload by decreasing vascular smooth muscle tone. Common genetic variants at the *NPPA *locus that are associated with the higher ANP concentration are also associated with lower blood pressure and reduced risk of hypertension [22]. In addition, *NPPA *expression is tightly regulated during the embryonic heart development [23,24], suggesting that complex regulatory mechanisms control the activity of *NPPA*.

## Results

### Natural antisense transcripts associated with candidate genes for blood pressure regulation

We first identified candidate genes [see Additional file 1 – Table S1] with the evidence of antisense transcription by screening them against published sense-antisense pairs [8] and scanning for expressed sequence tags (ESTs) on opposite strand using UCSC Genome Browser . We found that seven (*ADD3*, *NPPA*, *ATP1A1*, *NPR2*, *CYP17A1*, *ACSM3*, *SLC14A2*) out of 38 genes tested had multiexon NATs supported by a number of ESTs with canonical GT-AG splice donor-acceptor sites (Table 1). The antisense transcripts (indicated by suffix -*AS*, that stands for 'antisense') differ from each other in their organization and complementarity in respect to sense mRNA [see Additional file 2 – Figure S1]. The 5'-most exons of antisense transcript for adducin 3, *ADD3-AS*, have complementarity to 5' UTR exons of the sense gene (5'-5' overlap). In other cases, the overlap pattern is more complex, involving one or several internal exons and intron-exon boundaries. In two cases, the transcript from opposite strand has been annotated as a protein-coding mRNA: (i) *NPR2 *3'-most exon is partially complementary to *SPAG8 *[see Additional file 2 – Figure S1D] and (ii) *EXOD1 *is transcribed from opposite strand of *ACSM3 *[see Additional file 2 – Figure S1F]. In addition, the open reading frame (ORF) is predicted for one of the isoforms of *ADD3-AS *and *NPPA-AS *(99 and 121 amino acids, respectively). Taken together, these data indicate that antisense transcripts associated with blood pressure candidate genes have diverse structure and various relationships to their sense partners.

**Table 1:** Hypertension candidate genes associated with antisense transcripts.

**Gene**	Antisense overlap*	**Antisense mRNA/ESTs§**	**Antisense ORF**
***ADD3***(adducin 3) chr10:111,746,098-111,885,313	2 exons, both 5' UTR	U92992 (total 17 ESTs)	3 exons, 99 aa
***NPPA ***(natriuretic peptide precursor A) chr1:11,828,363-11,830,422	3 exons	BU732528 (total 12 ESTs)	1 exon, 121 aa
***ATP1A1 ***(Na+/K+ -ATPase alpha 1 subunit) chr1:116,717,359-116,748,919	3 coding exons	AK309389 (total 27 ESTs)	
***NPR2 ***(natriuretic peptide receptor B) chr9:35,782,406-35,799,728	1 exon (coding and 3' UTR)	*SPAG8 *(total 37 ESTs)	8 exons, 501 aa
***CYP17A1 ***(cytochrome P450, family 17) chr10:104,580,278-104,587,280	3 coding exons	BX100578 (total 6 ESTs)	
***ACSM3 ***(acyl-coenzyme A synthetase) chr16:20,682,813-20,715,980	1 coding exon	*EXOD1 *(total 49 ESTs)	11 exons, 328 aa
***SLC14A2 ***(solute carrier family 14, member 2) chr18:41,046,958-41,517,058	1 noncoding 5' UTR exon	AK126075 (total 12 ESTs)	

* Number of sense gene exons involved in sense-antisense complementarity. § A gene symbol or a GenBank accession of a representative EST or mRNA and a total number of ESTs in antisense cluster. ORF – open reading frame (only ORFs larger than 90 aa are shown), UTR – untranslated region, aa – amino acids.

### Structure and expression of *NPPA *in human tissues

**Figure 1:**
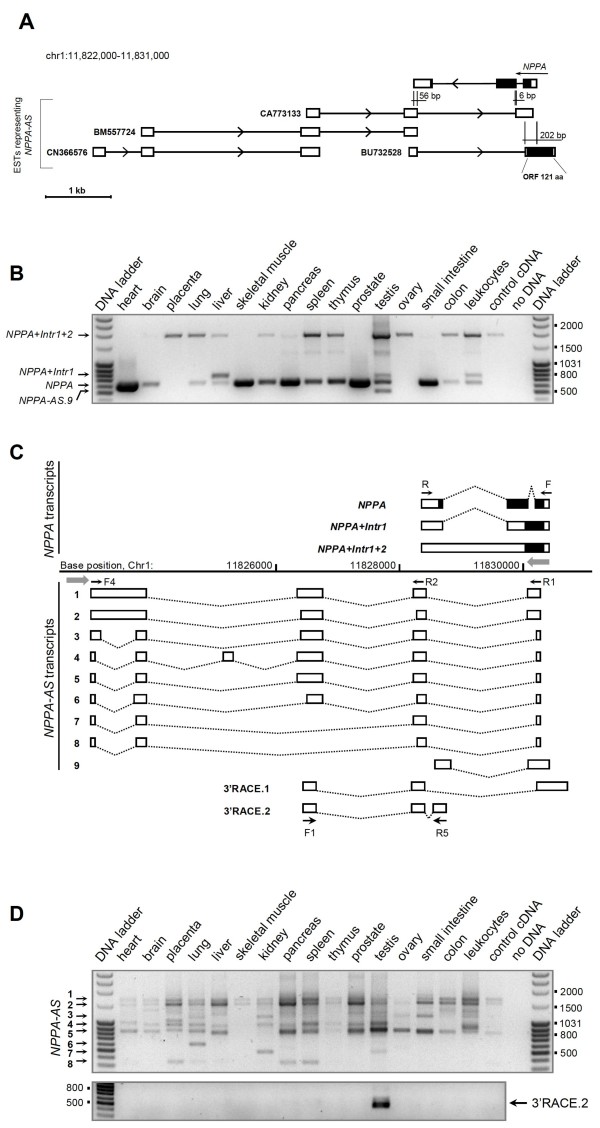
**Splicing isoforms and expression pattern of *NPPA *and *NPPA-AS***. (A) Genomic structure of *NPPA *and its antisense transcript, *NPPA-AS *(represented by several overlapping ESTs). Arrowheads indicate the direction of transcription, vertical lines depict the overlap between sense and antisense exons. The length of complementary region is shown in basepairs. Coding regions of *NPPA *and putative *NPPA-AS *ORF are represented as black boxes, noncoding exons and UTRs as white boxes. (B) The expression analysis of *NPPA *demonstrates the presence of intron-retained variants (*NPPA+Intr1+2*, 1820 bp and *NPPA+Intr1*, 722 bp) in a number of tissues. PCR reactions were performed using the primers NPPA-F and NPPA-R (indicated by arrows labeled F and R on Figure 1C) and resolved on an agarose gel. Expected size of correctly spliced *NPPA *product is 600 bp. The shorter product in testis (457 bp) represents a testis-specific isoform of antisense transcript *NPPA-AS.9*, which was not represented by ESTs in the databank and therefore could not be avoided when designing primers. (C) The schematic depiction of *NPPA *and *NPPA-AS *splicing isoforms, the products of 3' RACE reactions and their location on the genomic sequence. The gray block arrows indicate the direction of transcription. (D) *NPPA-AS *is widely expressed as a complex combination of alternatively spliced isoforms. Upper panel: the agarose gel showing the reactions performed using primers NPPA-AS-F4 and NPPA-AS-R1 (indicated by arrows labeled F4 and R1 on Figure 1C) on the panel of tissue-specific cDNAs. Lower panel: Isoform 3'RACE.2 is expressed only in testis. PCR was performed using primers NPPA-AS-F1 and NPPA-AS-R5 (indicated by arrows labeled F1 and R5 on Figure 1C). The band corresponds to the expected size of 405 bp.

For further studies we selected *NPPA *and its antisense transcript, *NPPA-AS *(Figure 1A). This sense-antisense pair was selected for functional studies because (i) *NPPA-AS *is represented by a cluster of alternatively spliced ESTs and overlaps with the exons, introns as well as with intron-exon boundaries of *NPPA*, raising the hypothesis that it could have a posttranscriptional regulatory role in *NPPA *expression and (ii) *NPPA *has a compact size, consisting of only three exons and spanning ~2 kb on human chromosome 1p36.22 that makes it a good model system for examination of the functional role of antisense transcription.

We investigated the expression of *NPPA *using commercial Human Multiple Tissue cDNA panels MTC I and II (Figure 1B, C). Consistent with previous studies [23-25], the strongest expression of *NPPA *was detected in heart, but several tissues contained additional alternative products. Sequencing of these products revealed that the largest *NPPA *band detected in many tissues (1820 bp) represents the isoform of *NPPA *with retained both introns (further referred to as *NPPA+Intr1+2*). Visual inspection of the agarose gel (Figure 1B) indicates that the expression of this unspliced form and correctly spliced *NPPA *appear to be inversely correlated. Alternative product of 722 bp that was observed in liver, testis and leukocytes contains the retained intron 1 (*NPPA+Intr1*). In addition, a product shorter than correctly spliced *NPPA *mRNA (457 bp) was detected in testis. Sequencing of this product revealed that its sequence and splicing pattern are similar to the transcripts originating from the opposite strand, suggesting that this is actually an isoform of *NPPA-AS*, which was not represented by any of the ESTs in the database. To ensure that the amplification of *NPPA+Intr1+2 *is not caused by genomic DNA contamination, additional PCR experiments were performed with several primers that detect only *NPPA-AS*, but not *NPPA *[see Additional file 2 – Figure S2]. Because *NPPA *and *NPPA-AS *are both transcribed from the same genomic locus, contamination with the genomic DNA should result in amplification of unspliced *NPPA-AS *as well. However, these reactions yielded only the products corresponding to correctly spliced *NPPA-AS*, indicating no presence of the genomic contamination and demonstrating that the *NPPA+Intr1+2 *isoform indeed represents mRNA with retained introns.

### *NPPA-AS *is expressed as a collection of alternatively spliced isoforms

Next, we characterized the structure and expression profile of *NPPA-AS *in human tissues using the panel of tissue-specific cDNAs (Figures 1C, D). Sequencing of eight identified isoforms confirmed that they all are spliced according to GT-AG consensus rule [see Additional file 2 – Figures S3 and S4]. It appears that *NPPA-AS *isoforms are not a result of alternative usage of different exons, but rather almost every exon displays at least two alternative splice donor/acceptor sites (Figure 1C). Majority of the alternative splicing events occur at the acceptor site of the intron. In addition, clearly identifiable polypyrimidine tract is located close to the splice acceptor site of all introns.

Next, we mapped the 3' end of *NPPA-AS *by RACE using RNA from HeLa cell line. We designed the gene-specific primers that would identify the 3' variants that are important in respect of complementarity with *NPPA*. Sequencing of the 3' RACE products identified two alternative 3' ends of *NPPA-AS*. One of the RACE products confirmed the presence of 3'-terminal exon that was predicted based on EST sequences CD368210 and BU732528 (3'RACE.1, Figure 1C). In addition, we identified a novel 3'-terminal exon that overlaps with the second intron and third exon of *NPPA *(3'RACE.2, Figure 1C). Expression analysis of the 3'RACE.2 isoform showed that among the sixteen tissues analysed, it is expressed only in testis (Figure 1D). Isoform 3'RACE.1 contains a suboptimal AGTAAA poly(A) signal 16 nucleotides upstream of the cleavage site, in the position where the majority of poly(A) signals are located [26]. 3'RACE.2 isoform does not contain a detectable polyadenylation signal, but an A-rich element (AAAGAGAACACAGACATA), similar to the element found in *PAPOLG *gene [27], that is also lacking any poly(A) signal variant, is located 19 nucleotides upstream of polyadenylation site. This suggests that in addition to alternative splicing, the processing of the *NPPA-AS *transcript might be regulated also at the level of polyadenylation.

### Primary sequence of *NPPA-AS *is not evolutionarily conserved

**Figure 2:**
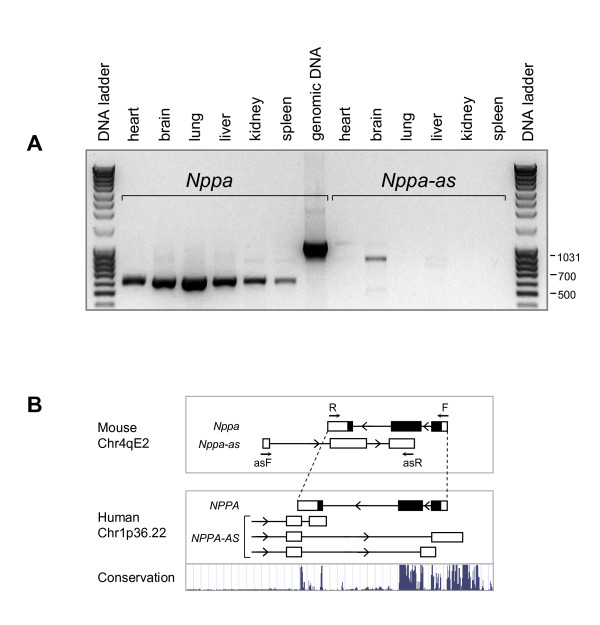
**Expression analysis of *Nppa *and *Nppa-as *in mouse tissues**. (A) *Nppa *is expressed in all tissues that were tested and *Nppa-as *is expressed in the brain. *Nppa *was amplified using primers mNppa-F and mNppa-R, *Nppa-as *was detected using primers mNppa-asF and mNppa-asR (indicated by arrows marked F, R, asF and asR on Figure 2B, respectively). (B) Genomic organization of *NPPA *locus in mouse and human. Arrowheads indicate the direction of transcription. Conservation profile of the human sequence, based on the UCSC vertebrate conservation track is shown below. Conservation is detected in the coding region (depicted as black boxes), promoter area and 3' terminus of *NPPA *gene, but not in the *NPPA-AS *exons that are located in introns or downstream of the *NPPA *gene.

To address the evolutionary conservation of *NPPA-AS*, we first asked whether *Nppa *gene in mouse is associated with similar natural antisense transcript. Two ESTs (GenBank:BQ771223 and CO043998 representing the 5' and 3' end of the IMAGE clone 6400656) at the *Nppa *locus originate from the opposite strand and therefore may represent *Nppa-as *in mouse. These ESTs are isolated from the brain of the mouse at embryonic day 12.5. Both ESTs follow the GT-AG splicing consensus, and CO043998 contains a canonical polyadenylation signal AATAAA as well as a short poly(A) tail. RT-PCR using RNA isolated from mouse tissues showed weak expression of *Nppa-as *in brain and very faint signal in liver (Figure 2A), while the expression of *Nppa *was present in all tissues that were tested (heart, brain, lung, liver, kidney and spleen) (Figure 2A). Exons of the mouse and human antisense transcript are not conserved at the primary structure level outside of the overlap regions with the *NPPA *exons and are located at the different positions in the genomic sequence (Figure 2B). However, both in mouse and in human, the antisense transcript overlaps with the intron-exon boundaries of *NPPA *gene (Figure 2B).

The last exon of *NPPA-AS *3'RACE.1 isoform (Figure 1C, represented also by ESTs GenBank:CD368210 and BU732528) contains an open reading frame (ORF) that is predicted to code for a protein of 121 amino acids [see Additional file 2 – Figure S5]. The multiple alignment and *in silico *translation demonstrate that in all organisms except human and chimpanzee, the predicted ORF is interrupted by at least one frameshift and one stop codon [see Additional file 2 – Figures S5A and S6]. Since the translated amino acid sequence does not contain any conserved domains and has no significant identity above 30% to any known protein, the function of the predicted protein in human and chimpanzee cannot be assessed based on the primary sequence.

### Positive correlation between the expression levels of intron-retained *NPPA *and specific *NPPA-AS *isoforms

**Figure 3:**
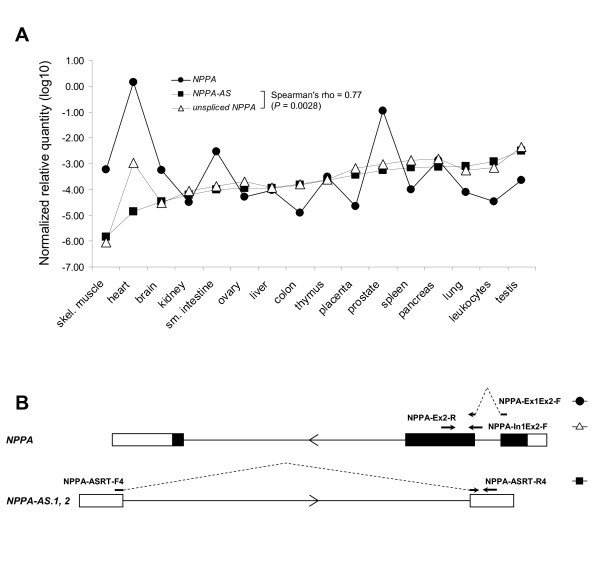
**Expression levels of *NPPA *variants with retained intron and *NPPA-AS *are highly correlated (Spearman's rank correlation coefficient = 0.77, p-value = 0.0028)**. (A) Expression levels of *NPPA-AS *(a total of isoforms 1 and 2) and *NPPA *(spliced and intron-retained forms were detected separately using primers shown on Figure 3B) were determined by quantitative real-time PCR on a panel of human tissue-specific cDNAs. Expression levels were normalized to endogenous *GAPDH *mRNA. (B) Schematic depiction of primer positions (black arrows) that were used to quantify the expression levels of *NPPA *and *NPPA-AS*. To the right are indicated the symbols that are used for each reaction in Figure 3A.

To study the correlation between the expression levels of *NPPA *and *NPPA-AS*, we quantified the specific *NPPA *and *NPPA-AS *isoforms using real-time PCR and commercial Human Multiple Tissue cDNA panels MTC I and II (Figure 3). We focused on the *NPPA-AS *isoforms *NPPA-AS.1 *and *2 *(Figure 3B) that are complementary to the first intron and first and second exon of *NPPA*, including intron-exon boundaries. These are the isoforms that most likely have an impact on *NPPA *expression regulation. We detected no correlation between the expression of correctly spliced *NPPA *and its antisense transcript (Figure 3A), but instead observed a strong positive correlation between the expression levels of *NPPA-AS *and *NPPA *variants with retained intron 1 (Spearman's rank correlation coefficient = 0.77, p-value = 0.0028). This result was confirmed by using an alternative set of primers detecting the same *NPPA-AS *isoforms and retained *NPPA *intron 1 (data not shown). Such correlation further indicates a possible functional relationship between *NPPA-AS *expression and posttranscriptional regulation of *NPPA*.

### *NPPA-AS *as a modulator of expression of *NPPA *splicing isoforms

**Figure 4:**
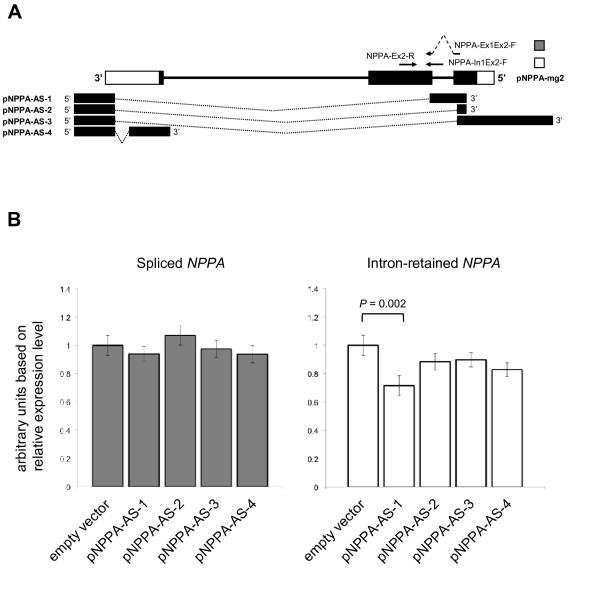
***NPPA-AS *constructs modulate the level of intron-retained *NPPA *variants**. (A) Schematic depiction of expression constructs and oligonucleotide primers that were used to quantify spliced *NPPA *(NPPA-Ex1Ex2-F and NPPA-Ex2-R) or the variants with retained intron 1 (NPPA-In1Ex2-F and NPPA-Ex2-R). (B) The expression of *NPPA *intron-retained form was reduced significantly in case of coexpression with pNPPA-AS-1. Mouse NIH3T3 cells were cotransfected with pNPPA-mg2 along with different *NPPA-AS *expression constructs depicted above or empty vector as a control. Relative expression levels of spliced (left panel) and intron-retained (right panel) *NPPA *mRNA were analysed by real-time quantitative PCR. The geometric mean of two reference genes, *GAPDH *and *HPRT1*, was used as an endogenous control. The results are represented as arbitrary units based on relative quantity at the logarithmic scale. Error bars show s.e.m. P-value was calculated using Mann-Whitney Test.

To test the hypothesis that *NPPA-AS *has a regulatory role in the splicing of *NPPA *mRNA, we constructed expression vectors containing either *NPPA *gene or one of the four antisense transcript isoforms with different regions of complementarity to *NPPA *(Figure 4A). Constructs pNPPA-AS-1, -2, -3 and -4 were generated by cloning of *NPPA-AS *isoforms *NPPA-AS.1 *and *2 *and3'RACE products 3'RACE.1 and 3'RACE.2, respectively, into the expression vector pQM-Ntag/A. Generation of different constructs expressing *NPPA *or *NPPA-AS *isoforms allows to test the effect of each antisense variant separately and eliminates the possible influence of transcriptional interference as a mechanism of antisense action.

We cotransfected *NPPA *expression construct into the mouse embryonic fibroblast cell line NIH3T3 in pairs with individual *NPPA-AS *constructs and quantified correctly spliced and intron-retained variants of *NPPA *by real-time RT-PCR. Mouse cell line was selected for transfection in order to eliminate the possible effect of endogenous expression of *NPPA *and *NPPA-AS*. The expression of specific *NPPA-AS *isoforms in transfected cells was confirmed by quantitative RT-PCR (data not shown). As shown in Figure 4B, expression level of intron-retained *NPPA *variant was significantly downregulated after transfection with pNPPA-AS-1 (*P *= 0.002, Mann-Whitney test). Although all constructs caused slight changes in expression levels of both spliced and intron-retained *NPPA*, the effects did no reach statistical significance in other experiments.

**Figure 5:**
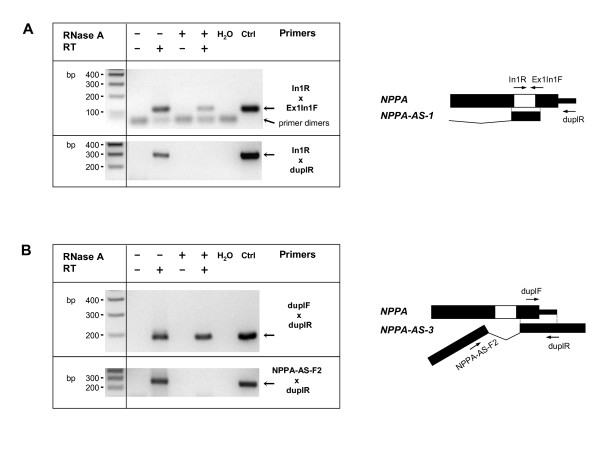
***NPPA-AS *forms RNA duplexes with *NPPA***. Reactions were performed with cloned intron-containing *NPPA *gene (pNPPA-mg2) and two antisense isoforms: pNPPA-AS-1 (A) and pNPPA-AS-3 (B). The agarose gels showing the results of PCR reactions using RNA samples treated (+) or non-treated (-) with RNase A. RT indicates reverse transcription reaction in the presence (+) or without (-) a reverse transcriptase. H_2_O, no template control; Ctrl, control reaction performed using cloned *NPPA *(A) or *NPPA-AS *(B) as a template.

To further test whether complementary sequences of *NPPA *and *NPPA-AS *actually can form RNA duplexes, we performed RT-PCR on RNase treated RNA samples from cotransfections of *NPPA *and *NPPA-AS *constructs pNPPA-AS-1 and 3 (Figure 5). In both cases, the product with the correct size from RNase-treated samples was obtained, indicating that *NPPA *and its antisense transcript indeed form duplex RNAs. pNPPA-AS-3 has complementarity only to exonic regions of *NPPA *and can form a duplex with both spliced and unspliced *NPPA *mRNA. In the case of pNPPA-AS-1 we detected the duplex formation with the intronic region of *NPPA*, which can occur only with unspliced pre-mRNA. These results indicate that *NPPA *and *NPPA-AS *interact at the RNA level and that a specific *NPPA-AS *isoform (*NPPA-AS.1*) can modulate the proportion of intron-retained *NPPA*.

## Discussion

The important regulatory role of endogenous noncoding RNAs, including antisense transcripts, has been proposed based on a number of large-scale transcription profiling studies. Because of the variety of functional mechanisms and lack of direct experimental support, the biological meaning of most of this noncoding transcription is still unclear. In the present study we have investigated *NPPA/NPPA-AS *sense-antisense transcript pair.

Several studies have addressed the question whether some fraction of antisense transcripts may in fact be artifacts of reverse transcription reaction [28,29]. To exclude such artifacts from our study, we considered only NATs with two or more exons and consensus splice sequences. In addition, 3' RACE reactions further confirmed the strand-specificity of *NPPA-AS *by identification of two alternative polyadenylated 3' terminal exons (Figure 1C).

*NPPA *is a functional candidate gene for elevated blood pressure, coding for an atrial natriuretic peptide (ANP), a member of a small family of endogenous peptide hormones. It is produced primarily by atrial cardiocytes in response to increasing cardiac wall tension. Association of specific *NPPA *variants with increased ANP levels as well as with lower blood pressure and reduced risk of hypertension [22] strongly support the central role of *NPPA *in the maintenance of blood pressure homeostasis. In addition, a region harbouring *NPPA *was among the eight loci identified in the meta-analysis of blood pressure genome-wide association studies [30]. Transcriptional regulation of *NPPA *and maturation of ANP have been studied quite extensively (for recent reviews see [31,32]) and our results add further evidence to the elaborate control of *NPPA *expression.

During the expression analysis of *NPPA *mRNA we found the strongest expression of correctly spliced *NPPA *in heart and moderate expression in a number of human tissues, including prostate, pancreas and small intestine, for example (Figure 1B). In addition, we observed the expression of *NPPA *isoforms with retained introns (*NPPA+Intr1 *and *NPPA+Intr1+2*) in several tissues (Figure 1B). Normally, ANP is synthesized as a 153-amino acid preprohormone. Removal of the signal peptide creates a 126-amino acid prohormone that is further cleaved to form a mature C-terminal 27-amino acid ANP. In the case of *NPPA+Intr1 *and *NPPA+Intr1+2*, the ORF that starts with the first methionine (Met-1) of prepro-ANP encodes only for a signal peptide region and is terminated after the frameshift caused by the intron retention. An alternative ORF of intron-retained *NPPA *isoforms, starting with the methionine Met-51 includes the mature ANP sequence, but since the peptide encoded by this putative ORF does not contain a signal sequence, its proper processing and biological activity is doubtful.

The natural antisense transcript of *NPPA*, that we named *NPPA-AS*, is widely present in human tissues and displays a complex pattern of alternative splicing (Figure 1D). All different *NPPA-AS *splicing isoforms overlap with both exonic and intronic regions of *NPPA*, including intron-exon boundaries (Figure 1C). Such overlap pattern raised the hypothesis that *NPPA-AS *may be involved in the regulation of *NPPA *expression.

Overlapping antisense gene pairs are preferentially co-expressed or inversely expressed in human tissues [6,33,34], supporting a model of negative control by antisense RNA that proposes state of balance in case of co-expression or up- or downregulation in case of inverse expression. Interestingly, the expression of *NPPA-AS *was strongly correlated with the intron-retained, rather than correctly spliced form of *NPPA *(Figure 3), indicating that it may play a functional role in posttranscriptional regulation of *NPPA *expression. Such correlation, however, does not necessarily indicate causal relationship, because it can be affected by many factors including regulation of transcription of both *NPPA *and *NPPA-AS*, or modulation of *NPPA *splicing by other factors.

Conservation often reflects functional significance of a nucleotide sequence. Among sense-antisense pairs, less than 7% are found to be conserved between human and mouse [35,36]. This may indicate that antisense transcripts are mostly species-specific, or alternatively, that the process of transcription, secondary structure or organization of the transcript rather than the primary sequence is functionally important. Among conserved sense-antisense pairs, about one third have identical expression pattern in mouse and human [37]. We found that expression profile in mouse and human is different for both *NPPA *and *NPPA-AS *(Figure 2). In mouse, *Nppa *is strongly expressed in some tissues (brain, lung, liver) where human *NPPA *is expressed weakly. We did not detect any alternatively spliced or intron-retained *Nppa *forms in mouse. In case of *NPPA-AS*, we found that neither the primary structure nor the expression pattern is conserved between mouse and human: in human it is expressed in all tissues examined, while in mouse the expression was observed only in brain (Figure 2A). However, both mouse and human antisense transcripts overlap with the exon-intron boundaries of *NPPA *(Figure 2B), implying that such genomic arrangement might be functionally significant.

Although many antisense transcripts overlap with the intron-exon boundaries of the sense mRNA, the effect of endogenous antisense transcripts as splicing regulators is studied in detail only in a few cases [16,38]. Modulation of mRNA splicing by exogenous antisense oligonucleotides has gained more attention and its therapeutic potential has been established in clinical trials involving patients with Duchenne's muscular dystrophy [39]. Much less is known about the role of endogenous antisense RNAs in regulation of splicing or stability of different mRNA isoforms. The complementarity of *NPPA *and its antisense exons suggests that if *NPPA-AS *has a function in regulation of *NPPA*, it depends on the mechanisms that involve the interactions at the RNA level. Our results show (Figure 4B) that at least one *NPPA-AS *isoform can modulate the ratio of unspliced and spliced *NPPA *variants, by decreasing the levels of intron-retained *NPPA *form. Since we were using minigene expression system, we excluded the effects of such possible regulatory mechanisms like transcriptional interference and heterochromatin formation [17]. Currently we do not know what is the exact mechanism of intron-retained *NPPA *downregulation, but it is possible that the formation of the duplex RNA due to the complementary regions can lead to post-transcriptional regulation via different mechanisms like RNA masking (in which case the binding of factors required for splicing or export is blocked), RNA editing or RNA interference [14]. Although the role of RNA interference in NAT-mediated regulation in mammals has been controversial, Watanabe *et al*. [14] identified seventeen loci in mouse where siRNAs arose from interaction of sense-antisense transcripts of the same locus. Considering the large number of NATs in mammals, the real extent of siRNA biogenesis via endogenous sense-antisense RNA interaction in mammalian cells remains still unknown. It is also possible that since antisense transcription extends through entire *NPPA *locus and into the promoter region, the mechanisms like transcriptional interference and modulation of *NPPA *promoter elements can occur and affect the expression of *NPPA *independently.

Although the biological role of *NPPA *antisense transcription needs further investigation, the regulatory role of *NPPA *in both adult cardiovascular system and in heart development during embryogenesis [23,24] suggest that *NPPA-AS *may be involved in fine-tuning of *NPPA *expression during embryonic development or in response to specific stimulus.

## Conclusion

We have identified the natural antisense transcripts of human blood pressure candidate genes and provide a detailed characterization of an antisense transcript associated with the *NPPA *gene. Our data support the biological significance of *NPPA-AS *by demonstrating that it (i) is widely expressed as a collection of canonically spliced isoforms, (ii) can directly interact with *NPPA *at the RNA level and (iii) is able to influence the levels of intron-retained *NPPA *variants.

## Methods

### Identification and *in silico *analysis of natural antisense transcripts

Candidate genes (n = 38) were selected according to the prior evidence of involvement in blood pressure regulation [see Additional file 1 – Table S1]. Most of the genes were selected based on the published data on the biology and genetics of blood pressure regulation. The selection included also genes responsible for the Mendelian forms of hypertension or hypotension, location near linkage peaks or quantitative trait loci (QTLs), reports on animal models and human association studies. Additional information was obtained from different resources (OMIM, ; NCBI GeneBank and NCBI Locuslink ; Ensembl ). Candidate gene list was also supplemented with loci involved in other cardiovascular diseases like myocardial infarction, coronary artery disease and stroke. The candidate genes were screened for antisense transcripts using recently published sense-antisense transcript data [8]. The structure and direction of the transcripts was verified using UCSC Genome Browser . To avoid random „transcriptional noise” and genomic DNA contamination, we considered only transcripts with at least two exons, canonical splice sites (GT/AG) and overlap with at least one exon of the sense gene. In addition, several transcripts had a polyadenylation signal and poly(A) tail, supporting together with canonical splice sites their strand-specificity.

Multiple alignment of sequences corresponding to predicted ORF of *NPPA-AS *from different species was performed using ClustalW2 at . Sequence database searches were performed using BLAST programs at .

### PCR and sequencing

Expression analysis of *NPPA *and *NPPA-AS *was carried out using Human Multiple Tissue cDNA panels MTC I and II (BD Biosciences) and primers [see Additional file 1 – Table S2] that were designed using Primer3 program . *G3PDH *primers were included with MTC panels. PCR on mouse tissue-specific cDNAs was performed using oligonucleotides designed according to mouse *Nppa *gene and EST BQ771223 (for *Nppa-as*). PCR conditions were: 75 mM Tris-HCl (pH 8.8), 20 mM (NH_4_)_2_SO_4_, 0.01% Tween 20, 2.5 mM MgCl_2_, 250 μM dNTPs and 2.5 u per 100 μl Taq DNA Polymerase (Fermentas). Cycling conditions followed the touch-down procedure, namely initial denaturation at 94°C for 2 m, followed by 11 cycles at 94°C for 30 s, annealing for 30 s at temperatures decreasing from 62 to 57°C (with 0.5°C decremental in each cycle), 72°C for 60 s, and 30 cycles at 94°C for 30 s, 57°C for 30 s, 72°C for 60 s, and ending with an extension step at 72°C for 5 m.

For sequencing, PCR products were extracted from agarose gel using NucleoSpin Extract II (Macherey-Nagel) and either cloned into the pTZ57R vector using InsT/Aclone Kit (Fermentas) or sequenced directly after ExoI/SAP (both Fermentas) treatment. Sequencing reactions were performed using BigDye Terminator v3.1 Cycle Sequencing Kit (Applied Biosystems) according to manufacturer's instructions and analyzed on ABI Prism™ 3730xl DNA Analyzer. Sequencing results were manually analyzed using Bioedit software  and mapped to the genome using BLAT alignment tool [40] at the UCSC Genome Browser . Novel sequences obtained in this study have been submitted to GenBank database (FJ706070–FJ706079).

### 3' RACE (rapid amplification of cDNA ends)

The 3' RACE reactions were performed using GeneRacer™ Kit (Invitrogen) and 1 μg of total RNA isolated from semi-confluent HeLa cells grown in 5% CO2 at 37°C. Reverse transcription was performed using SuperScript III Reverse Transcriptase and GeneRacer™ Oligo dT Primer (both included in the kit). Amplification was carried out using GeneRacer™ or 3' primer and the following gene-specific primers: NPPA-AS-F1, -F2 or -F5. Nested PCR was performed using nested GeneRacer™ primer and suitable gene-specific primers.

### Construction of plasmids

The vector pQM-Ntag/A (Quattromed, Estonia) was used to create expression constructs under the control of CMV promoter. *NPPA *gene was amplified from human genomic DNA using primers NPPA-GFXba and NPPA-GRBam and inserted into the *Xba*I and *Bam*HI sites of pQM-Ntag/A. These primers amplify a 2069 bp fragment (chr1:11828358-11830426 according to human genome assembly hg18) of genomic DNA, including all three *NPPA *coding exons and both UTRs. Four constructs representing different *NPPA-AS *splicing isoforms were generated. pNPPA-AS-1 and -2 represent splicing isoforms NPPA-AS.1 and NPPA-AS.2 obtained with oligonucleotides NPPA-AS-F4 and NPPA-AS-R1. For generation of pNPPA-AS-3 and -4, 3' RACE products 3'RACE.1 and 3'RACE.2 were utilized. All constructs were sequenced using internal primers as well as universal primers flanking the insert cloning site.

### Transfection of expression constructs

Cells were grown in 5% CO2 at 37°C and on the day before transfection were plated into 24-well plates using medium without antibiotics. Next day, cells were transfected using Lipofectamine™ 2000 reagent (Invitrogen) and 1.0 μg of total plasmid DNA (0.2 μg of pNPPA-mg2 and 0.8 μg of any of the *NPPA-AS *constructs or empty vector). Cells were incubated at 37°C and RNA was isolated 24 hours later. For relative quantitation experiments, transfections were carried out in triplicate three times (total nine replicates).

### RNA isolation

Total RNA from cell lines and from mouse (male C57bl/6) tissues was isolated using TRIzol reagent (Invitrogen) and the quantity and quality of RNA was assessed using Nanodrop ND-1000 (Thermo Scientific). DNase treatment was performed using TURBO DNA-free Kit™ (Ambion) with 1 μg of RNA in a volume of 30 μl. cDNA was synthesized using 2 μg of total RNA and First Strand cDNA Synthesis Kit (Fermentas) according to manufacturer's instructions.

### Real-time quantitative PCR

For investigation of expression levels of *NPPA *and *NPPA-AS *in human tissues, the Human Multiple Tissue cDNA panels MTC I and II (BD Biosciences) were used. Amplification was performed with the following primer pairs: NPPA-Ex1Ex2-F and NPPA-Ex2-R for quantification of spliced *NPPA*; NPPA-In1Ex2-F and NPPA-Ex2-R for detection of unspliced *NPPA*. For quantification of *NPPA-AS*, primers NPPA-ASRT-F4 and NPPA-ASRT-R4 were used. *GAPDH *was used as an endogenous control and amplified with primers GAPDH-S and GAPDH-AS. For quantification of spliced and unspliced *NPPA *from transfection experiments, RNA from mouse NIH3T3 cells was isolated and DNase-treated as described above. Primers were designed avoiding binding to mouse sequences to prevent nonspecific amplification. Correctly spliced *NPPA *mRNA was detected using primers NPPA-Ex1Ex2-F and NPPA-Ex2R, unspliced *NPPA *was detected using primers NPPA-In1Ex2-F and NPPA-Ex2-R. Two endogenous reference genes were used: *GAPDH *(amplified with primers GAPDH-S and GAPDH-AS) and *HPRT1 *(amplified with primers HPRT1-S and HPRT1-AS). The reactions were performed in the 96-well microtiter plate using ABI PRISM^® ^7900 Real-Time PCR cycler. The 25 μl reaction mixture consisted of 3 μl of 1:10 cDNA dilution, 12.5 μl of ABsolute™ QPCR SYBR^® ^Green ROX Mix (Thermo Scientific), 70 nM forward and reverse primer. The cycling parameters were: enzyme activation at 95°C for 15 m followed by 40 cycles 95°C for 15 s, 60°C for 30 s, 72°C for 30 s. Reactions were performed in triplicates for each biological replicate. As negative controls for DNA contamination, reactions without the reverse transcriptase were carried out. We performed control and optimization experiments for all primer pairs and selected for actual quantitation experiments primer pairs with amplification efficiency of 100 ± 10%. During optimization, serial dilutions of template were used and the specificity of the PCR products was confirmed by the presence of a single peak during the dissociation curve analysis. Amplification efficiency of the reactions was 100 ± 10% and intra- and inter-assay variation coefficients were below 3% and 8%, respectively. PCR efficiencies were calculated from ten-fold dilution series and relative expression of correctly spliced and intron-retained *NPPA *was calculated according to Pfaffl [41] by taking PCR efficiency into account. The geometric mean of *GAPDH *and *HPRT1 *was used as an endogenous control. Statistical significance of the results was analyzed using two-tailed Mann-Whitney test.

### Duplex detection

RNA from NIH3T3 cells cotransfected with constructs pNPPAmg2 and pNPPA-AS-1 or pNPPA-AS-3 was isolated as described previously and treated with DNase (TURBO DNA-free Kit™, Ambion) according to manufacturer's instructions and 0.5 μl of RNase A (10 mg/ml, Fermentas). RNase was inactivated with 0.5 mg/ml proteinase K treatment at the presence of 1% SDS for 30 min at 37°C. RNA was extracted with phenol/chloroform treatment and cDNA was synthesized using primers In1R (for NPPA-AS-1 duplex detection) or duplR (for NPPA-AS-3) and First Strand cDNA Synthesis Kit (Fermentas). In parallel, control experiments using RNAs not treated with RNase A and reverse transcriptions without reverse transcriptase were performed. PCR was performed with primers In1R and Ex1In1F for detection of *NPPA:*:*NPPA-AS-1 *duplex and with primers duplF and duplR for detection of *NPPA:*:*NPPA-AS-3 *duplex.

## Authors' contributions

TA and ML designed the study, KK and ML selected and analysed the candidate genes, TA performed the experiments and carried out the analyses, TA and ML wrote the paper. All authors read and approved the final manuscript.

## Supplementary Material

Additional file 1 **Candidate genes for blood pressure regulation**. The file contains the detailed list of blood pressure regulation candidate genes that were screened for the presence of natural antisense transcripts and oligonucleotide primer sequences used in this study. Click here for file

Additional file 2 **The genomic structure of natural antisense transcripts**. Additional information describing in detail the genomic structure of natural antisense transcripts and expression analysis and multiple alignment of *NPPA-AS*. Click here for file
