# Supplementary material for: Natural antisense transcript of natriuretic peptide precursor A (NPPA): structural organization and modulation of NPPA expression
Source: BMC Mol Biol. 2009 Aug 11;10:81. doi: 10.1186/1471-2199-10-81 (PMC2731763; doi:10.1186/1471-2199-10-81)
Supplement: Additional file 1 — Candidate genes for blood pressure regulation. The file contains the detailed list of blood pressure regulation candidate genes that were screened for the presence of natural antisense transcripts and oligonucleotide primer sequences used in this study. [file 1471-2199-10-81-S1.doc]

**Additional file 1**

**Table S1.** Description of candidate genes for blood pressure regulation. Genes associated with antisense transcripts are shown in bold. Chromosomal position is indicated according to Human 2006 (hg18) assembly. Gene function and disease association was obtained from OMIM and LocusLink databases at NCBI (http://www.ncbi.nlm.nih.gov/).

| **Gene symbol** | **Gene name** | **Locuslink ID** | **Chromosomal location** | **Gene function** | **Disease/Syndrome** |
| --- | --- | --- | --- | --- | --- |
| *ACE* | angiotensin I converting enzyme | 1636 | chr17:58908166-58928711 | Converts angiotensin I to angiotensin II by release of the terminal HIS-LEU, this results in an increase of the vasoconstrictor activity of angiotensin. | Susceptibility to myocardial infarction. |
| ***ACSM3*** | acyl-coenzyme A synthetase | 6296 | chr16:20682813-20715980 | SA (rat hypertension-associated) homolog; medium-chain fatty acid:CoA ligase activity with broad substrate specificity (in vitro). Acts on acids from C(4) to C(11) and on the corresponding 3-hydroxy- and 2,3- or 3,4- unsaturated acids (in vitro). | Hypertension, essential. |
| *ADD1* | adducin 1 | 118 | chr4:2815382-2901587 | Membrane-cytoskeleton-associated protein that promotes the assembly of the spectrin-actin network. Binds to calmodulin. | Essential salt-sensitive hypertension, hyperlipidemia familial combined, huntington disease, stroke, renal disease, insulin resistance, coronary heart disease. |
| *ADD2* | adducin 2 | 119 | chr2:70742771-70848837 | Membrane-cytoskeleton-associated protein that promotes the assembly of the spectrin-actin network. Binds to calmodulin. Calmodulin binds preferentailly to the beta subunit. | Essential salt-sensitive hypertension, hereditary elliptocytosis and spherocytosis, hemolysis, anemia. |
| ***ADD3*** | adducin 3 | 120 | chr10:111746098-111885313 | Membrane-cytoskeleton-associated protein that promotes the assembly of the spectrin-actin network. | Essential salt-sensitive hypertension. |
| *AGT* | angiotensinogen preproprotein | 183 | chr1:228904892-228916564 | In response to lowered blood pressure, the enxyme renin cleaves angiotensin I, from angiotensinogen. | Hypertension, essential, susceptibility to, pregnancy-induced hypertension (PIH) (Preeclampsia). |
| *AGTR1* | angiotensin II receptor, type 1 | 185 | chr3:149898348-149943480 | Receptor for angiotensin II. Mediates its action by association with G proteins that activate a phosphatidylinositol - calcium second messenger system. | Hypertension, essential; renal disease, heart failure; cardiovascular diseases; nephropathy obstructive. |
| *AGTR2* | angiotensin II receptor, type 2 | 186 | chrX:115216031-115219848 | Receptor for angiotensin II. May have role in cell morphogenesis and related events in growth and development. | Hypertension, essential; renal disease, heart failure, cardiovascular diseases; nephropathy obstructive. |
| ***ATP1A1*** | Na+/K+ -ATPase alpha 1 subunit | 476 | chr1:116717359-116748919 | This is the catalytic component of the active enzyme, which catalyzes the hydrolysis of ATP coupled with the exange of Na and K ions across theplasma membrane. This action creates the electrochemical gradient of Na and K, providing the energy for active transport of various nutrients. | Hypertension. |
| *BSND* | barttin | 7809 | chr1:55237205-55247053 | Functions as a beta-subunit for CLCNKA and CLCNKB chloride channels. In the kidney CLCNK/BSND heteromers mediate chloride reabsorption by facilitating its basolateral efflux. In the stria, CLCNK/BSND channels drive potassium secretion by recycling chloride for the basolateral SLC12A2 cotransporter. | Bartter syndrome, gitelman syndrome, hypokalemia, hypotension, renal failure. |
| *CLCNKA* | chloride channel Ka | 1187 | chr1:16221073-16233132 | Voltage-gated chloride channel. | Bartter syndrome, type 4, digenic. |
| *CLCNKB* | chloride channel Kb | 1188 | chr1:16242939-16256063 | Voltage-gated chloride channel. | Bartter syndrome, antenatal. |
| *CYP11B1* | cytochrome P450, subfamily 11B, polypeptide 1 | 1584 | chr8:143950775-143958238 | Has steroid 11-beta-hydroxylase activity. In addition to this activity, the 18 or 19-hydroxylation of steroids and the aromatization of androstendione to estrone have also been ascribed to cytochrome P450 XIB. | Adrenal hyperplasia, congenital, due to 11-beta-hydroxylasedeficiency, aldosteronism, glucocorticoid-remediable. |
| *CYP11B2* | cytochrome P450, subfamily 11B, polypeptide 2 | 1585 | chr8:143988977-143996261 | Catalyzes the conversion of 11-deoxycorticosterone to aldosterone via corticosterone and 18-hydroxycorticosterone. | Hypoaldosteronism, congenital, due to CMO II deficiency, hypoaldosteronism, congenital, due to CMO I deficiency, low renin hypertension, aldosterone to renin ratio raised. |
| ***CYP17A1*** | cytochrome P450, subfamily 17A, member 1 | 1586 | chr10:104580278-104587280 | Conversion of pregnenolone and progesterone to their 17-alpha-hydoxylated products and seubsequently to dehydroepiandrosterone (DHEA) and androstenedione. Catalyzes both the 17-alpha-hydroxylation and the 17, 20-lyase reaction. Involved in sexual development during fetal life and at buberty. | Adrenal hyperplasia, congenital, due to 17-alpha-hydroxylasedeficiency. |
| *GNB3* | guanine nucleotide-binding protein, beta-3 | 2784 | chr12:6819379-6826818 | Guanine nucleotide-binding proteins (G proteins) are involved as a modulator or transducer in various transmembrane signaling systems. The beta and gamma chains are required for the GTPase activity, for replacement of GDP by GTP, and for G protein-effector interaction. | Hypertension, essential, susceptibility to. |
| *HSD11B1* | 11-beta-hydroxysteroid dehydrogenase 1 | 3290 | chr1:207926173-207974918 | Catalyzes reversibly the conversion of cortisol to the inactive metabolite cortisone. | Cortisone reductase deficiency. |
| *HSD11B2* | 11-beta-hydroxysteroid dehydrogenase 2 | 3291 | chr16:66022537-66028955 | Has a role in modulating glucocorticoid activity both at the level of the mineralcorticoid receptor and the glucocorticoid receptor. Uses NADH while 11-DH1 uses NADPH. Catalyzes non reversibly the conversion of cortisol to the inactive metabolite cortisone. | Apparent mineralocorticoid excess, hypertension due to, hypertension, mild low-renin. |
| *KCNJ1* | potassium inwardly-rectifying channel J1 | 3758 | chr11:128213125-128242478 | In the kidney, probably palys a major role in K+ homeostasis. Iward rectifier K+ channels are characterized by a greater tendancy to allow potassium to flow into the sell rather than out of it. Their voltage dependance is regulated by yhe concentration of extracellular potassium; as external K+ is rased, the voltage range of the channel opening shifts to more positive voltages. the inward rectification is mainly due to the blockage of outward current by internal magnesium. this channel is activated by internal ATP and can be blocked by external BA2+. | Bartter syndrome type 2, characterized by an hypokalemic,hypochloremic metabolic alkalosis with hyperkaliury, hyperexcretion of prostaglandin E,hyperreninemia hyperaldosteronism with normal blood pressure, insensitivity to AGT2, and hyperplasia of juxtaglomerular apparatus, autosomal recessive. |
| *KLK1* | kallikrein 1 preproprotein | 3816 | chr19:56014216-56018855 | Glandural kallikreins cleave Met-Lys and Arg-Ser bonds in kininogen to release Lys-bradykinin. | Renal failure chronic. |
| *NEDD4L* | ubiquitin ligase NEDD4-like | 23327 | chr18:53862778-54216369 | E3 ubiquitin-protein ligase which accepts ubiquitin from an E2 ubiquitin-conjugating enzyme in the form of a thioester and then directly transfers the ubiquitin to targeted substrates. Inhibits TGF-beta signaling by triggering SMAD2 and TGFR1 ubiquitination and proteasome-dependent degradation. | Liddle syndrome, essential hypertension, hypotension orthostatic. |
| ***NPPA*** | natriuretic peptide precursor A | 4878 | chr1:11828363-11830422 | Atrial natriuretic factor (ANF) is a potent vasoactive substance synthesized in mammalian atria and is thought to play a key role in cardiovascular homeostasis. Has a CGMP-stimulating activity. | Heart failure, heart failure congestive, essential hypertension, cardiac hypertrophy. |
| *NPPB* | natriuretic peptide precursor B | 4879 | chr1:11840108-11841579 | Acts as a cardiac hormone with a variety of biological actions including natriuresis, diuresis, vasorelaxation, and inhibition of renin and aldosterone secretion. It is thought to play a key role in cardiovascular homeostasis. Helps restore the body´s salt and water balance. Improves heart function. | Heart failure, heart failure congestive, heart diseases; cardiac overload. |
| *NPPC* | natriuretic peptide precursor C | 4880 | chr2:232498379-232499203 | Vasorelaxant activity. Has a CGMP-stimulating activity. | Heart failure, heart failure congestive, cardiovascular diseases. |
| *NPR1* | natriuretic peptide receptor 1 | 4881 | chr1:151917737-151933088 | Receptor for atrial natriuretic peptide. Has guanylate cyclase activity on binding of ANF. | Cardiac hypertrophy, heart failure congestive, essential hypertension, heart failure. |
| ***NPR2*** | natriuretic peptide receptor B | 4882 | chr9:35782406-35799728 | Receptor for atrial natriuretic peptide. Has guanylate cyclase activity on binding of ANF. Seems to be stimulated more effectively by brain natriuretic peptide (BNP) than by ANP. | Acromesomelic dysplasia Maroteaux type (AMDM); heart failure, heart failure congestive, essential hypertension. |
| *NR3C2* | nuclear receptor subfamily 3, group C, member 2 | 4306 | chr4:149219370-149582973 | Receptor for both mineralcorticoids (MS) such as aldosterone and glucocorticoid (GS) such as cortisone or cortisol. The effect of MC is to increase ion and water transport and thus raise extracellular fluid volume and blood pressure and lower potassium levels. | Pseudohypoaldosteronism type I, autosomal dominant. Hypertension, early-onset, autosomal dominant, with exacerbation in pregnancy. |
| *REN* | renin precursor | 5972 | chr1:202390571-202402088 | Renin is a highly specific endopeptidase, whose only known function is to generate angiotensin I from angiotensinogen in the plasma, initiating a cascade of reactions that produce an elevation of blood pressure and increased sodium retention by the kidney. | Hyperproreninemia. |
| *SCNN1A* | sodium channel, nonvoltage-gated 1 alpha | 6337 | chr12:6326274-6354976 | Sodium permeable non-voltage-sensitive ion channel inhibited by the diuretic amiloride. Mediate the electodiffusion of the luminal sodium (and water, which follows osmotically) through the apical membrane of epithelial cells. Controls the reabsorption of sodium in kidney, colon, lung and sweat glends. | Pseudohypoaldosteronism, type I. |
| *SCNN1B* | sodium channel, nonvoltage-gated 1, beta | 6338 | chr16:23221092-23300121 | Sodium permeable non-voltage-sensitive ion channel inhibited by the diuretic amiloride. Mediate the electodiffusion of the luminal sodium (and water, which follows osmotically) through the apical membrane of epithelial cells. Controls the reabsorption of sodium in kidney, colon, lung and sweat glands. Also plays role in taste perception. | Liddle syndrome, Pseudohypoaldosteronism, type I. |
| *SCNN1G* | sodium channel, nonvoltage-gated 1, gamma | 6340 | chr16:23101541-23135701 | Sodium permeable non-voltage-sensitive ion channel inhibited by the diuretic amiloride. Mediate the electodiffusion of the luminal sodium (and water, which follows osmotically) through the apical membrane of epithelial cells. Controls the reabsorption of sodium in kidney, colon, lung and sweat glands. Also plays a role in taste perception. | Liddle Syndrome, pseudohypoaldosteronism type 1. |
| *SGK1* | serum/glucocorticoid regulated kinase 1 | 6446 | chr6:134532081-134537695 | Protein kinase that plays an important role in cellular stress response. Activates certain potassium, sodium, and chloride channels, suggesting an involvement in the regulation of processes such as cell survival, neuronal excitability, and renal sodium excretion. Sustained high levels and activity may contribute to conditions such as hypertension and diabetic nephropathy. | Essential hypertension, cardiac hypertrophy, renal failure chronic. |
| *SLC12A3* | solute carrier family 12, member 3 | 6559 | chr16:55456643-55504850 | Electrically silent transporter system. Mediates sodium and chloride reabsorption. | Gitelman syndrome characterized by and hypokalemic alkalosis Bartter-like associated with hypocalciuria and hypomagnesemia. |
| ***SLC14A2*** | solute carrier family 14, member 2 | 8170 | chr18:41448764-41517058 | Specialized low-affinity urea transporter. Mediates urea transport in kidney. | Hypertension. |
| *SLC22A2* | solute carrier family 22, member 2 | 6582 | chr6:160512083-160599949 | Solute carrier family 22,member A2,polyspecific transporter oforganic cations,mainly expressed in kidney. | Cardiomyopathy; renal failure, hodgkin disease. |
| *SLC8A1* | solute carrier family 8, member 1 | 6546 | chr2:40192790-40532713 | Rapidly transporting Ca2+ during excitation-contraction coupling. Ca(2+) is extruded from the cell during relaxation SO as to prevent overloading of intracellular stores. | Heart failure, arrhythmia, ischemia, cardiomyopathy, essential hypertension, hypertension arterial, cardiovascular diseases. |
| *WNK1* | WNK lysine deficient protein kinase 1 | 65125 | chr12:732993-888219 | Controls sodium and chloride ion transport by inhibiting the activity of WNK4, potentially by either phosphorylating the kinase or via an interaction between WNK4 and the autoinhibitory domain of WNK1. WNK4 regulates the activity of the thiazide- sensitive Na-Cl cotransporter, SLC12A3, by phosphorylation. | Pseudohypoaldosteronism, type IIC, essential hypertension. |
| *WNK4* | WNK lysine deficient protein kinase 4 | 65266 | chr17:38186222-38202610 | Regulates the activity of the thiazide-sensitive Na-Cl cotransporter, SLC12A3, by phosphorylation which appears to prevent membrane trafficking of SLC12A3. Also inhibits the renal K(+) channel, KCNJ1, via a kinase-independent mechanism by which it induces clearance of the protein from the cell surface by clathrin-dependent endocytosis. WNK4 appears to act as a molecular switch that can vary the balance between NaCl reabsorption and K(+) secretion to maintain integrated homeostasis. | Pseudohypoaldosteronism type II. |

**Table S2.** Oligonucleotide primer sequences.

| **Oligonucleotide name** | **Sequence** |
| --- | --- |
|  | |
| Detection and cloning of *NPPA* isoforms | |
| NPPA-F | AGAGCAGCAAGCAGTGGATT |
| NPPA-R | CCATGGCAACAAGATGACAC |
| NPPA-GFXba | GCATCTAGACAGACGTAGGCCAAGAGAGG |
| NPPA-GRBam | GCTGGATCCATGGTGCTGAAGTTTATTC |
|  | |
| Detection and cloning of *NPPA-AS* isoforms | |
| NPPA-AS-F1 | CCAGTTCAGCTCCTGCATC |
| NPPA-AS-F2 | AACGCAGGCATTTGTCTTCT |
| NPPA-AS-F3 | GCTCAGAGGATCTTGGCATC |
| NPPA-AS-F4 | ATTCCCAGTCACCCACTCAC |
| NPPA-AS-F5 | TGGGGAATCCATTCTGTTTC |
| NPPA-AS-R1 | CCAGAGCTAATCCCATGTAC |
| NPPA-AS-R2 | TCAGGCCGATATTCCTTGTC |
| NPPA-AS-R3 | ATGGATGCAGGAGCTGAACT |
| NPPA-AS-R4 | CAGTGGTCACTGGCTCAGAA |
| NPPA-AS-R5 | AAGCCAGATATGTCTGTGTTCTC |
|  | |
| Detection of mouse *Nppa* and *Nppa-as* | |
| mNppa-F | GGCAGAGACAGCAAACATCA |
| mNppa-R | ACAGTGGCAATGTGACCAAG |
| mNppa-asF | AGGGCAACAGTGGACAGTCT |
| mNppa-asR | GGTACTGGGTCCATTCCTGA |
|  |  |
| Detection of *NPPA::NPPA-AS* RNA duplexes | |
| duplF | ACGGCATTGTACATGGGATT |
| duplR | CAGACGTAGGCCAAGAGAGG |
| Ex1In1-F | AGACCTGATGGATTTCAAGGTA |
| In1-R | CACTGACTTGGAGGAAATCAAG |
|  |  |
| Real-time quantitative PCR primers | |
| NPPA-In1Ex2-F | CTGTATTTTCCTTTTCTAAAGAATT |
| NPPA-Ex1Ex2-F | AGACCTGATGGATTTCAAGAATT |
| NPPA-Ex2-R | GGCACGACCTCATCTTCTAAA |
| NPPA-ASRT-F4 | CACTTTCAGTAACAGCAAATTC |
| NPPA-ASRT-R4 | TTTCCTCCAAGTCAGTGAGG |
| HPRT1-S | GTTAAGCAGTACAGCCCCAAAATG |
| HPRT1-AS | AAATCCAACAAAGTCTGGCCTGTA |
| GAPDH-S | TGTCAAGCTCATTTCCTGGTATGA |
| GAPDH-AS | CTTACTCCTTGGAGGCCATGTAG |
