# Supplementary material for: Natural antisense transcript of natriuretic peptide precursor A (NPPA): structural organization and modulation of NPPA expression
Source: BMC Mol Biol. 2009 Aug 11;10:81. doi: 10.1186/1471-2199-10-81 (PMC2731763; doi:10.1186/1471-2199-10-81)
Supplement: Additional file 2 — The genomic structure of natural antisense transcripts. Additional information describing in detail the genomic structure of natural antisense transcripts and expression analysis and multiple alignment of NPPA-AS. [file 1471-2199-10-81-S2.pdf]

Additional file 2

Figure S1. Genomic structure of six natural antisense transcripts that are associated with hypertension candidate genes in human. The structure of *NPPA* and *NPPA-AS* is shown on Figure 1A in the main text. Chromosomal position according to Human Mar. 2006 (hg18) assembly is shown. Arrows indicate the direction of transcription. Antisense transcripts are identified by appendix "*–AS*", except for antisense partners for *NPR2* and *ACSM3*, that are indicated according to GenBank annotations as *SPAG8* and *EXOD1*, respectively. In case where alternative splicing isoforms could be predicted from ESTs, they are numbered and shown separately. Overlap between sense and antisense exons is indicated in basepairs. Nucleotide sequence at the splice sites of antisense transcripts is shown (except for *SPAG8* and *EXOD1*), with uppercase letters indicating exonic and lowercase letters indicating intronic sequence.

A

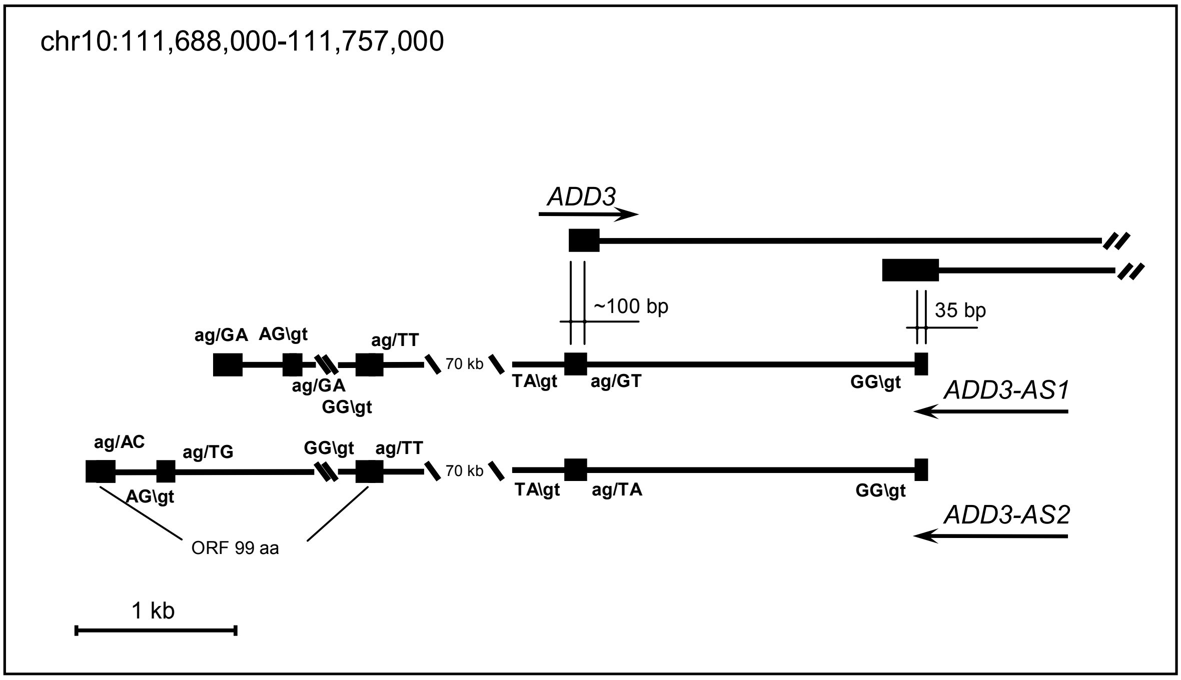

B

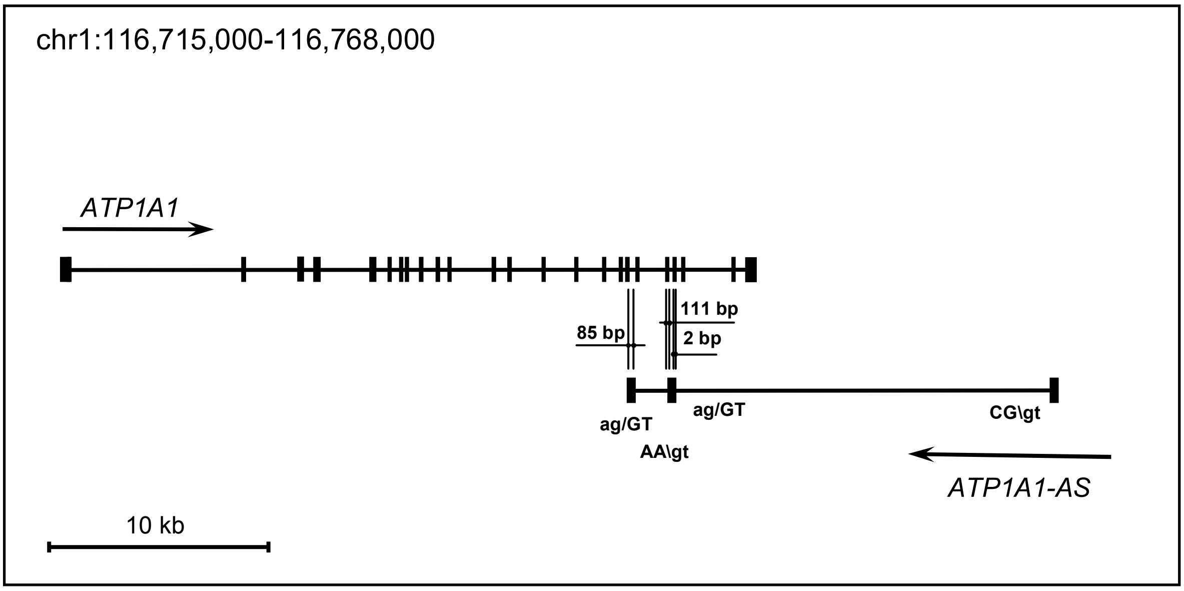
chr1:116,715,000-116,768,000
ATP1A1
111 bp
85 bp
2 bp
ag/GT
ag/GT
CG\gt
AA\gt
ATP1A1-AS
10 kb

C

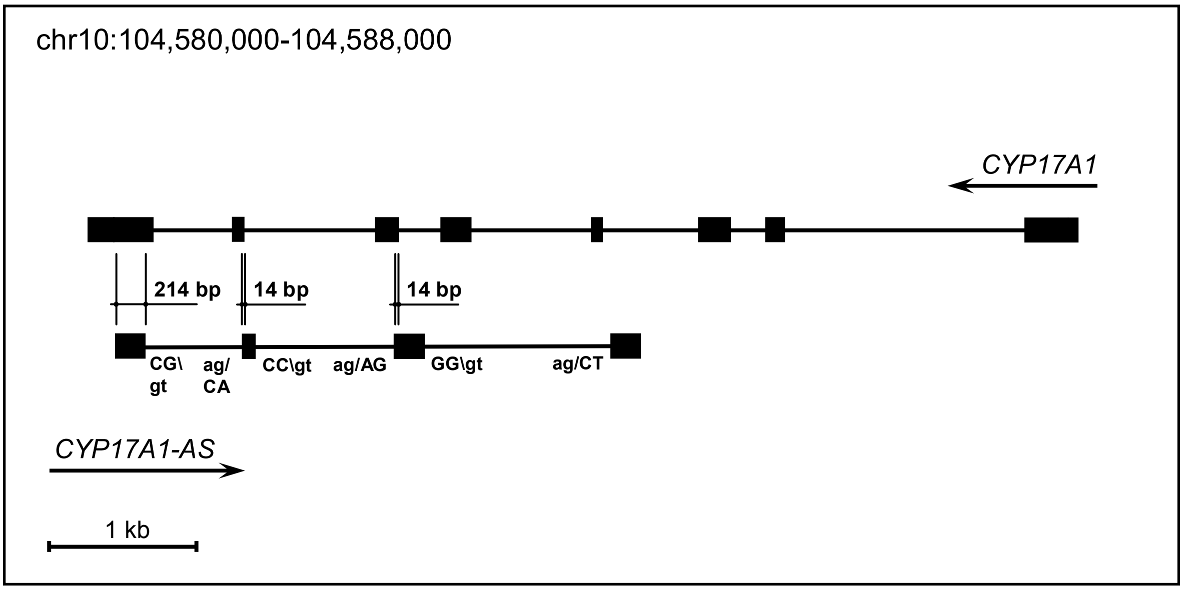
chr10:104,580,000-104,588,000
CYP17A1
214 bp
14 bp
14 bp
CG\gt
ag/CA
CC\gt
ag/AG
GG\gt
ag/CT
CYP17A1-AS
1 kb

D

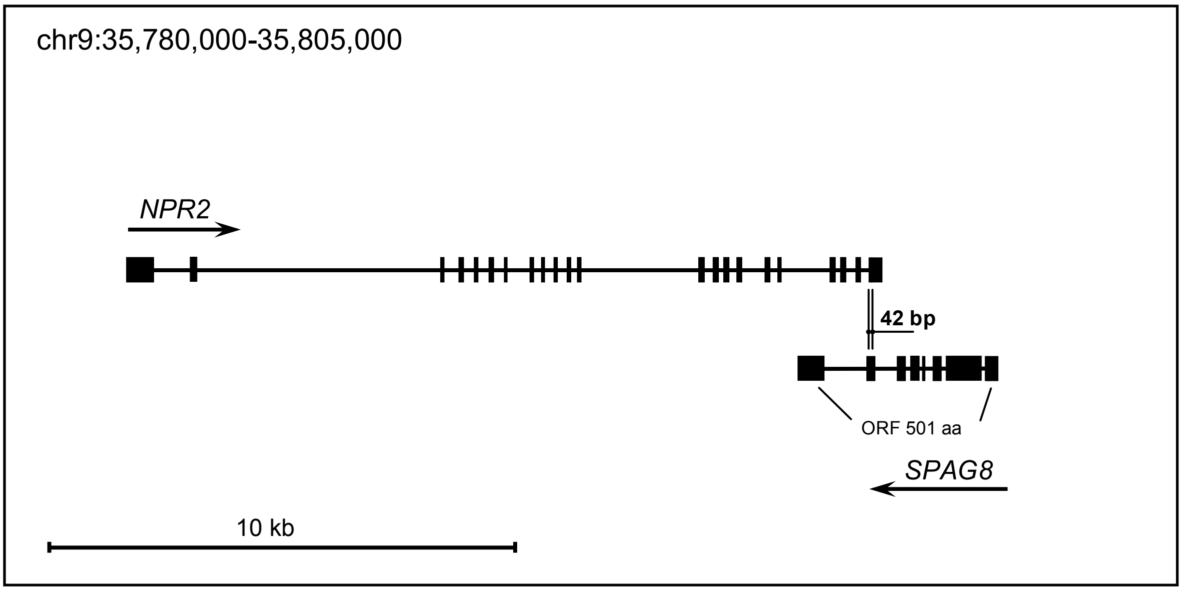
chr9:35,780,000-35,805,000
NPR2
42 bp
ORF 501 aa
SPAG8
10 kb

E

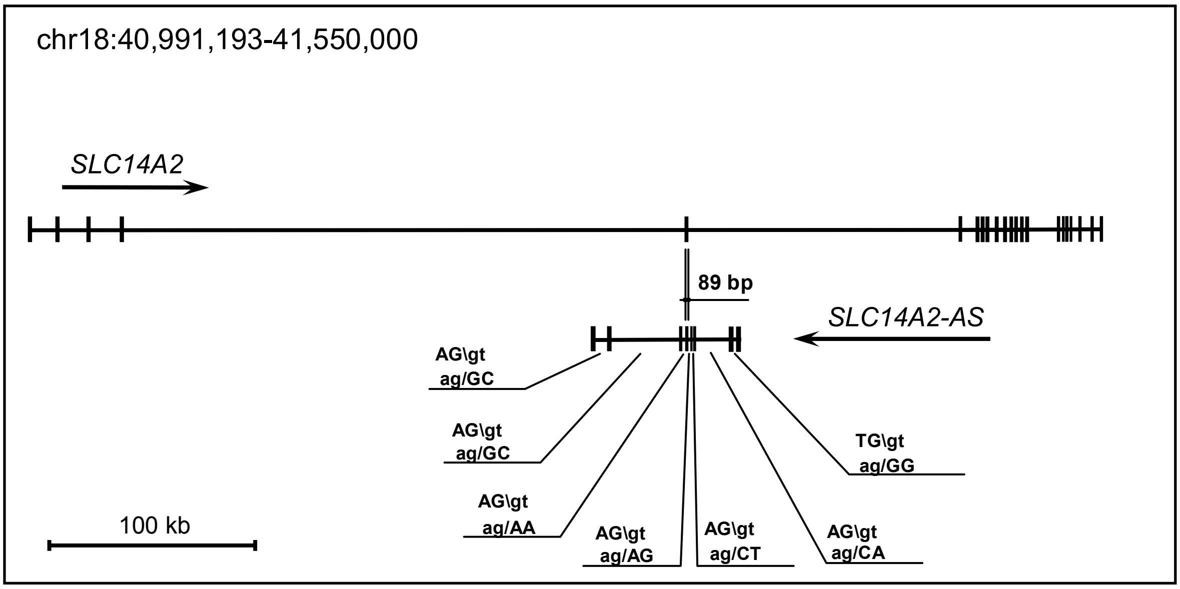
chr18:40,991,193-41,550,000
SLC14A2
89 bp
SLC14A2-AS
AG\gt ag/GC
AG\gt ag/GC
AG\gt ag/AA
AG\gt ag/AG
AG\gt ag/CT
AG\gt ag/CA
TG\gt ag/GG
100 kb

F

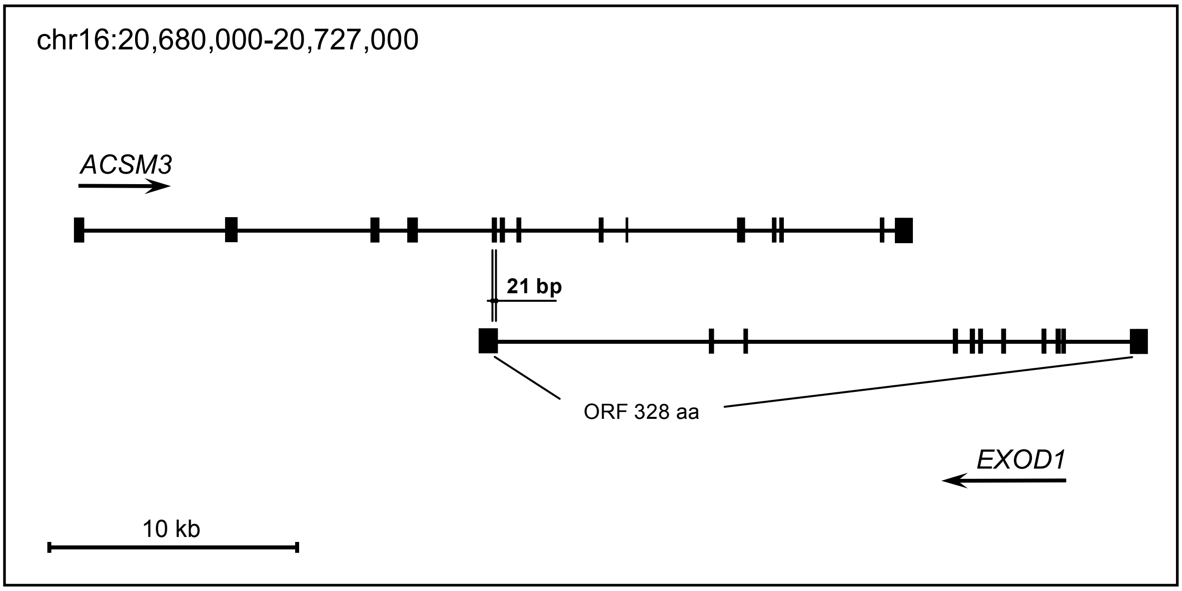

Figure S2. Detection of *NPPA-AS* expression using oligonucleotides NPPA-AS-F1 and NPPA-AS-R1 (A), NPPA-AS-F1 and NPPA-AS-R2 (B). Location of primers is shown in Figure 1. Primer sequences are provided in Table S2, Additional file 1. Expected products of 296, 439 bp (panel A) and 156 (panel B) were observed on the agarose gel. No unspliced products were detected, excluding the possibility that intron-containing *NPPA* form in Figure 1 derived from genomic DNA contamination.

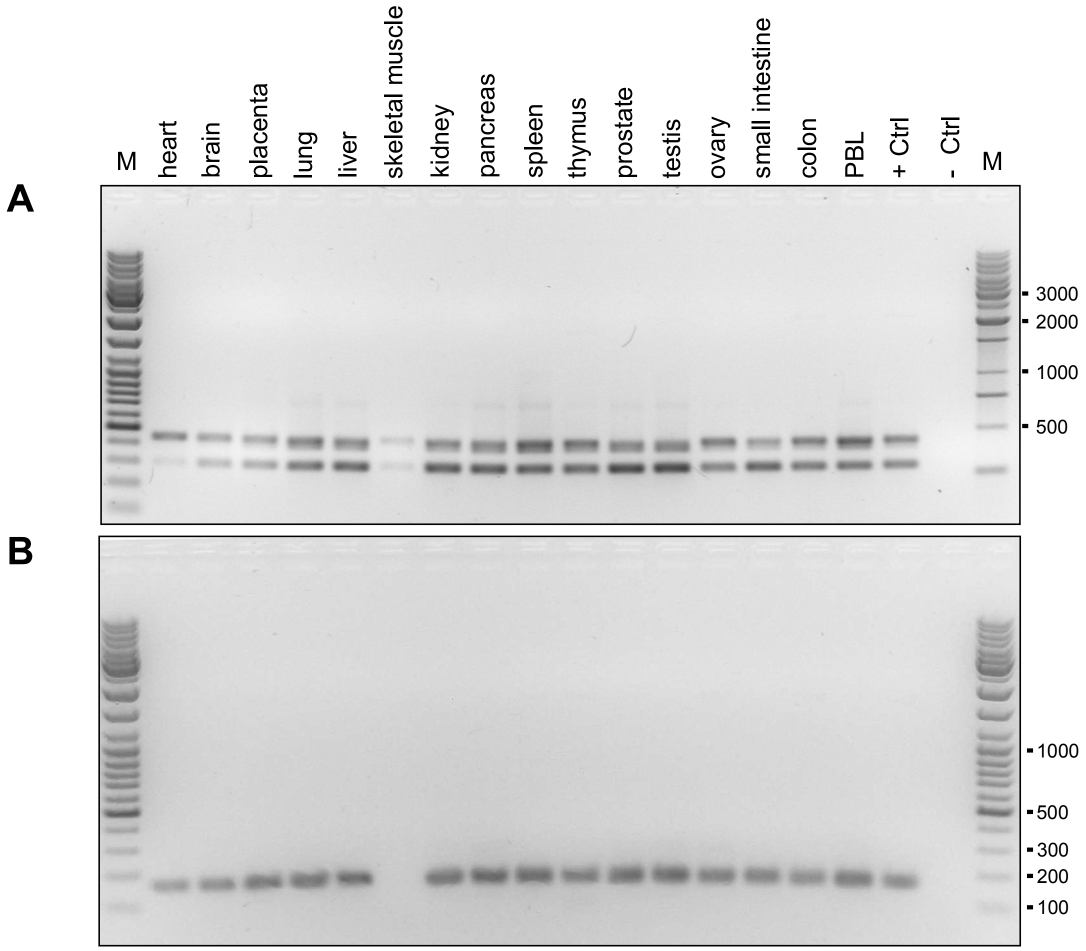

Figure S3. The length (in nucleotides) of *NPPA-AS* alternatively spliced exons from Figure 1B. Each exon is labeled using a combination of letters and numbers. Exact coordinates and sequence of exons is shown in Figure S4.

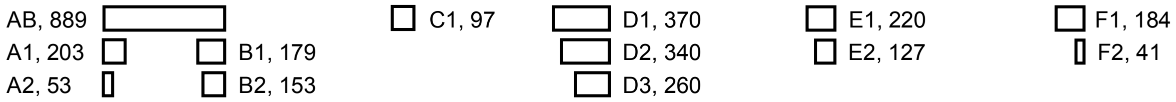

Figure S4. Nucleotide sequence of *NPPA-AS* exons. Nucleotide positions are indicated according to Human March 2006 (hg18) assembly. In addition to exonic sequence (uppercase), ten nucleotides of flanking intronic sequence (lowercase) for each exon is included (except for first and last exon, where the PCR primers were located).

```
>NPPA-AS_exon-AB_889bp (chr1:11822963-11823861)
ATTCCCAGTC ACCCACTCAC TCAGAAAGCC GGGAGTCATC GGACACCTTG  11823012
CTGGTCAGAG GTCCTGGGGG TGGTTTTGAA CCATCAGAGC TTGGACTTTT  11823062
CTGACTTCCC CAGCAAGGAT CTTCCCACTT CCTGCTCCCT GTGTTCCCAC  11823112
CCTCCAGTGT TGGCACAGGC CCACCCCTGG CTCCACCAGA GCCAGAAGCA  11823162
GAGGTAGAAT CAGGCGGGCC CCGGGCTGCA CTCCGAGCAG TGTTCCTGGC  11823212
CATCTTTGCT ACTTTCCTAG AGAACCCGGC TGTTGCCTTA AATGTGTGAG  11823262
AGGGACTTGG CCAAGGCAAA AGCTGGGGAG ATGCCAGTGA CAACATACAG  11823312
TTCATGACTA GGTTTAGGAA TTGGGCACTG AGAAAATTCT CAATATTTCA  11823362
GAGAGTCCTT CCCTTATTTG GGACTCTTAA CACGGTATCC TCGCTAGTTG  11823412
GTTTTAAGGG AAACACTCTG CTCCTGGGTG TGAGCAGAGG CTCTGGTCTT  11823462
GCCCTGTGGT TTGACTCTCC TTAGAACCAC CGCCCACCAG AAACATAAAG  11823512
GATTAAAATC ACACTAATAA CCCCTGGATG GTCAATCTGA TAATAGGATC  11823562
AGATTTACGT CTACCCTAAT TCTTAACATT GCAGCTTTCT CTCCATCTGC  11823612
AGATTATTCC CAGTCTCCCA GTAACACGTT TCTACCCAGA TCCTTTTTCA  11823662
TTTCCTTAAG TTTTGATCTC CGTCTTCCTG ATGAAGCAGG CAGAGCTCAG  11823712
AGGATCTTGG CATCACCCAC CAAAGTTAGC TGAAAGCAGG GCACTCCTGG  11823762
ATAAAGCAGC TTCACTCAAC TCTGGGGAAT GCTACCATTT TTTTTCCAAA  11823812
GTAGAAAGGA AGCACTTCTG AGCCAGTGAC CACTGAAAGg tatgtgcta

>NPPA-AS_exon-A1_203bp (chr1:11822953-11823175)
ggcatggaag  11822962
ATTCCCAGTC ACCCACTCAC TCAGAAAGCC GGGAGTCATC GGACACCTTG  11823012
CTGGTCAGAG GTCCTGGGGG TGGTTTTGAA CCATCAGAGC TTGGACTTTT  11823062
CTGACTTCCC CAGCAAGGAT CTTCCCACTT CCTGCTCCCT GTGTTCCCAC  11823112
CCTCCAGTGT TGGCACAGGC CCACCCCTGG CTCCACCAGA GCCAGAAGCA  11823162
GAGgtagaat cag

>NPPA-AS_exon-A2_53bp (chr1:11822953-11823025)
ggcatggaag  11822962
ATTCCCAGTC ACCCACTCAC TCAGAAAGCC GGGAGTCATC GGACACCTTG  11823012
CTGgtcagag gtc

>NPPA-AS_exon-B1_179bp (chr1:11823663-11823861)
tttccttaag  11823672
TTTTGATCTC CGTCTTCCTG ATGAAGCAGG CAGAGCTCAG AGGATCTTGG  11823722
CATCACCCAC CAAAGTTAGC TGAAAGCAGG GCACTCCTGG ATAAAGCAGC  11823772
TTCACTCAAC TCTGGGGAAT GCTACCATTT TTTTTCCAAA GTAGAAAGGA  11823822
AGCACTTCTG AGCCAGTGAC CACTGAAAGg tatgtgcta

>NPPA-AS_exon-B2_153bp (chr1:11823689-11823861)
cctgatgaag  11823698
CAGGCAGAGC TCAGAGGATC TTGGCATCAC CCACCAAAGT TAGCTGAAAG  11823748
CAGGGCACTC CTGGATAAAG CAGCTTCACT CAACTCTGGG GAATGCTACC  11823798
ATTTTTTTTC CAAAGTAGAA AGGAAGCACT TCTGAGCCAG TGACCACTGA  11823848
AAGgtatgtg cta

>NPPA-AS_exon-C1_97bp (chr1:11825155-11825271)
tgtccattag  11825164
AAACCAGCCT TTTCAGCATC TCACCCATTA GCAGCCCCAT CACCCAGTGA  11825214
TCAGTCGCCT CAGTAAAGCA GATCTGTGGA TGGGGAGCCT ACGGGTGgta  11825264
agaaagtg

>NPPA-AS_exon-D1_370bp (chr1:11826338-11826727)
```

```
ctcccttcag  11826347
ATGAACACTC TTCCTGATCC TCTCCTCTAG AATTCATCTC CTCCTGCTAG  11826397
CAGCCGCGTC CTGGAGGAGC AGCGGATGGG GAATCCATTC TGTTTCTTCC  11826447
TGGTGTTTAG GAAGTTGCCC CACACACAGA TTGCCCCGAT GTCCAACCAG  11826497
AAGAAGTGAA ACTGCTGCTG GGTCTGGAGA GGTGAAGACC CGTGGCCAGC  11826547
TTCTGTTGTT GCCATCGGCC ATTGCTTTTT GTTCGCTTGC TTTTGGTTTT  11826597
GCAAGAAGAG CGGCCTCTGT CTCTGATCTG CTTCAAATCA TCATTCCATC  11826647
AGTGACAGAA GTGGCTGTTC CATCAGTGGT CGCAGCCAGT TCAGCTCCTG  11826697
CATCCATCCC CAAGTGTTCT gtaagtccca

>NPPA-AS_exon-D2_340bp (chr1:11826368-11826727)
tctcctctag  11826377
AATTCATCTC CTCCTGCTAG CAGCCGCGTC CTGGAGGAGC AGCGGATGGG  11826427
GAATCCATTC TGTTTCTTCC TGGTGTTTAG GAAGTTGCCC CACACACAGA  11826477
TTGCCCCGAT GTCCAACCAG AAGAAGTGAA ACTGCTGCTG GGTCTGGAGA  11826527
GGTGAAGACC CGTGGCCAGC TTCTGTTGTT GCCATCGGCC ATTGCTTTTT  11826577
GTTCGCTTGC TTTTGGTTTT GCAAGAAGAG CGGCCTCTGT CTCTGATCTG  11826627
CTTCAAATCA TCATTCCATC AGTGACAGAA GTGGCTGTTC CATCAGTGGT  11826677
CGCAGCCAGT TCAGCTCCTG CATCCATCCC CAAGTGTTCT gtaagtccca

>NPPA-AS_exon-D3_260bp (chr1:11826448-11826727)
tggtgtttag  11826457
GAAGTTGCCC CACACACAGA TTGCCCCGAT GTCCAACCAG AAGAAGTGAA  11826507
ACTGCTGCTG GGTCTGGAGA GGTGAAGACC CGTGGCCAGC TTCTGTTGTT  11826557
GCCATCGGCC ATTGCTTTTT GTTCGCTTGC TTTTGGTTTT GCAAGAAGAG  11826607
CGGCCTCTGT CTCTGATCTG CTTCAAATCA TCATTCCATC AGTGACAGAA  11826657
GTGGCTGTTC CATCAGTGGT CGCAGCCAGT TCAGCTCCTG CATCCATCCC  11826707
CAAGTGTTCT gtaagtccca

>NPPA-AS_exon-E1_220bp (chr1:11828181-11828420)
accccactag  11828190
GAGTGGAATT TGAGGCCTCC CCAACCACCT ACCAAAAAAG GAGGGTGAAA  11828240
TGAAAGGAAG AAGAAAAACT CAGCATTCTT TCCTCTGACA AAGAGTAAAA  11828290
CGACAAGGAA TATCGGCCTG AATTCTCTTC CCAAGAAGAA AGAAAGCACA  11828340
CCAACGCAGG CATTTGTCTT CTGTCCATGG TGCTGAAGTT TATTCACTTT  11828390
CAAACCACTT TCAGTAACAG gtgaggttct

>NPPA-AS_exon-E2_127bp (chr1:11828274-11828420)
tctgacaaag  11828283
AGTAAAACGA CAAGGAATAT CGGCCTGAAT TCTCTTCCCA AGAAGAAAGA  11828333
AAGCACACCA ACGCAGGCAT TTGTCTTCTG TCCATGGTGC TGAAGTTTAT  11828383
TCACTTTCAA ACCACTTTCA GTAACAGgtg aggttct

>NPPA-AS_exon-F1_184bp (chr1:11830068-11830261)
aatggtccag  11830077
CAAATTCTTT AGAAAAGGAA AATACAGGGA AAGGGATAAA CCTCACTGAC  11830127
TTGGAGGAAA TCAAGAGGAG TGAGCACAGC ATCAGAAAGC CCCCTGGCCC  11830177
CAGACTGCAC CCGCTTTCCT GGCCCTACCT TGAAATCCAT CAGGTCTGCG  11830227
TTGGACACGG CATTGTACAT GGGATTAGCT CTGG

>NPPA-AS_exon-F2_41bp (chr1:11830211-11830261)
aatccatcag  11830220
GTCTGCGTTG GACACGGCAT TGTACATGGG ATTAGCTCTG G  11830260
```

Figure S5. Analysis of *NPPA-AS* ORF. (A) The predicted ORF in human (121 aa) is disrupted by several frameshift mutations and stop codons in different mammalian species. In all cases, degree of identity is higher at the nucleotide than at the amino acid level, indicating the nonconserved nature of the ORF. On top, VISTA conservation plot is shown for mouse, rat, dog, cow and opossum. Below, the percent of identity, the number of frameshifts and stop codons interrupting the predicted ORF is shown for following organisms: Ptr, *Pan troglodytes;* Mma, *Macaca mulatta*; Bph, *Balaenoptera physalus* (fin whale); Cfa, *Canis familiaris*; Mmu, *Mus musculus*; Rno, *Rattus norvegicus*; Bta, *Bos taurus*; Oar, *Ovis aries*; Mdo, *Monodelphis domestica* (gray short-tailed opossum). (B) Predicted amino acid sequence of a putative protein encoded by the *NPPA-AS* ORF.

A

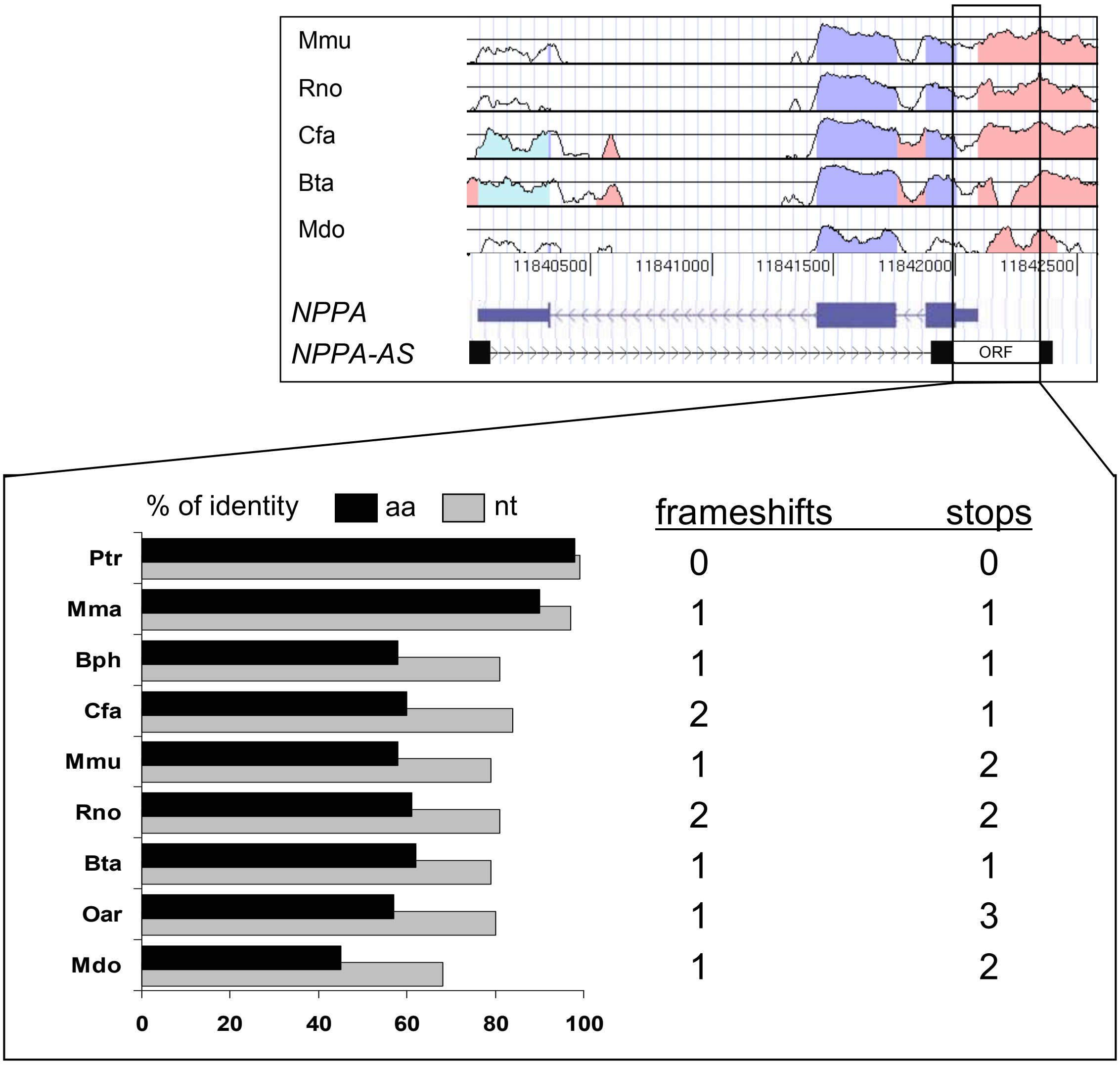

B

>Hsa-NPPA-AS_predicted_ORF
MLASSRSNPLLAALSLPSGSSLVPLSWPTSVPVSQLPSAASFYSPPALQARRRDRTLPILSLAVIKLPSE
KPAGDALLKLSSRRACHSPSLPWAAPTAPQTLSCKSRILTDLTQEAPTSKV*

Figure S6. Multiple sequence alignment of human *NPPA-AS* ORF (Figure S5) with respective regions from different mammalian species. Alignment was performed using ClustalW2 (http://www.ebi.ac.uk/Tools/clustalw2/index.html). Asterisks indicate nucleotide positions that are identical in all species. Hsa, *Homo sapiens*; Ptr, *Pan troglodytes* (chimpanzee); Mma, *Macaca mulatta* (rhesus macaque); Cja, *Callithrix jacchus* (marmoset); Bph, *Balaenoptera physalus* (fin whale); Mmu, *Mus musculus* (mouse); Fca, *Felis catus* (cat); Cfa, *Canis familiaris* (dog); Eca, *Equus caballus* (horse); Bta, *Bos taurus* (cow).

```
Hsa    ATGCTGGCGTCG-TCAAGGAGCAATCCACTGCTTGCTGCTCTGTCTCTCCCCTCTGGTTC 59
Ptr    ATGCTGGCGTCG-TCAAGGAGCAATCCACTGCTTGCTGCTCTGTCTCTCCCCTCTGGTTC 59
Mma    ATGCTGGCGTAG-TCAAGGAGCAATCCACTGCTTGCTGCTCTGTCTCTCCCCTCTGGTTC 59
Cja    ATGCTGGTGTAG-TCAAGGAGCGATCGACTGCTTGCTGCTCTGTCTCTCCCCTCTGGTTC 59
Bph    ATGCTGGCGTCGGTCAAGGGG-------CGGTCTGGTGCTTGCTGCCTCTTCTCTC--TC 51
Mmu    ATGCTGGCGTGGGTCGGGGCACGATCTGATGTTTGCTG-TCTCTGC--CCACTCTGGTTT 57
Fca    ATGATGGCGTCGGTCAAGGAG-------TGTTCTGATGCTTGCTG---CCTCTCTG--TC 48
Cfa    ATGCTGGCGTCGCTCGAGGAG-------TGGCCCGGTGCTTGCTG---CTCCCCTG--TC 48
Eca    ATGCTGGCGTCGGTCAAGGAG---------GTCTGGTGCTTGCTGCCTCTTCCCTC--TC 49
Bta    ATGCTGGCGTCGGTCAAGGAATGGT---CGGTCTGGTGCTTGCTGCCTCCTCCCTG--TC 55
       *** *** *  * **  **               * ** *   *    *  * **   *

Hsa    CTCTCTGGTTCCCCTCTCTTGGCCTACGTCTGTCCCTGTCTCCCAGCTGCCCAGTGCCGC 119
Ptr    CTCTCTGGTTCCCCTCTCTTGGCCTATGTCTGTCCCTGTCTCCCAGCTGCCCAGTGCCTC 119
Mma    CTCTCTGGTTCCCCTCTCTTGGCCTACGTCCGTCCCTGTCTCCCAGCTGCCCAGTGCCTC 119
Cja    CTCTCTAGTTCCCCTCTCCTGGCCTACGTCCGTCCCTGTCTCCAAACTGCCCAGCACCTC 119
Bph    CTCTCTGCTTTCCCACCCTCGGCCTCCCTCCGTCTCCGTCTCCCAGCTGCCCTGCGCCTC 111
Mmu    CTTTCT---CTCTCTCTCTCAGCTTTTGTCCGTCACTGTCTCCTAGCTGCC-AGCATCTC 113
Fca    CTCTC--------TTCTCTCAGCCTCCCTCCGCCT--GTCTCCCAGCTGCCCTGCGCCTC 98
Cfa    CTCTCG--CCTCCCTCTCTCAGCCTCCGTCTGCCTCTGTCTCCCAGCTGCCCTGCGCCTC 106
Eca    CTCCCTGGTTTCCCTCTCTTGGCGTTCGTCTGTCTCTGTCTCCCAGCTGCCCTGCGCCTC 109
Bta    CTCTTTGGCCTTTTGCTGTTGGCCTTTGTCCGTCTCTGTCTCCCAGCTGCCCTGAGCCTC 115
       **             *     ** *   ** * *   ****** * *****  *   * *

Hsa    CTCTTTTTATAGCCC-CCCAGCTCTCCAAGCACGCA------GGAGAGACAGAACCCTCC 172
Ptr    CTCTTTTTATAGCCC-CCCAGCTCTCCAAGCACGCA------GGAGAGACAGAACCCTCC 172
Mma    CTCTTTTTATAGCCC-CCCAGCTCTCCAAGCACGCA------GGAGAGACAGAACCCTCC 172
Cja    CTCTTTTTATAGTCCTCCCAACTCTTCAAGCACGCA------GGAAAGATAGAACCCTCC 173
Bph    CTCTTTTTATAGCCC-CTTGGCTCTCTGAGAGCACA------AGAGGGACGGAACCCTCC 164
Mmu    CCGTTTTTATAGCCC-CCCAGCTCTTTGAGAAGGCA------GGGGGGCTAGAACCCTCC 166
Fca    CTCTTTTTATAGCCC-CCCAGCTCTCCAAGAGCGCA------AGAGGGACGGAACCCTCC 151
Cfa    CTCTTTTTATAGCCC-CCCGGCTCTCCAAGAGCACA------AGAGGGACGGAACCCTCC 159
Eca    CTCTTTTTATAGCCC-CCCAGCTCTCCGAGAACGCA------AGAGGGATGGAACCCTCC 162
Bta    CTCTTTTTATAGCCC-CCTGGCTCTCTGGGAGCTCGTCCGGGAGAGGGACGGAACCCTCC 174
       *  ********* ** *    ****    *    *            *   *********

Hsa    CCATTCTGTCACTTGCAGTGATAAAGCTGCCAAGTGAGAAGCCAGCAGGAGATGCCCTTT 232
Ptr    CCATTCTGTCACTTGCAGTGATAAAGCTGCCAAGTGAGAAGCCAGCAGGAGATGCCCTTT 232
Mma    CCATTCTGTCACTTGCAGCGATAAAGCAGCCAAGTGAGAAGCCAGCAGGAGATGCCCTTT 232
Cja    CCATTCTGTCACTTGCAGTGATAAAGCAGCCGAGAGAGAAGCCAGCAGGAGATGCCCTTT 233
Bph    CCATTCTGTCACTTGCAGCGATAAAGCAGCTGAGTGAGGAGCCAGCAGAAGGTGCCCTTT 224
Mmu    CCATTCTGTCACTTGCAGCGATAAAGCGATGGAGTGAGGAGCCAGCAGAAGATGCCCTTT 226
Fca    CCATTCTGTCACTTGCAGCGATAAAGCAGCTGAATGAGGGAACAGCAGAAGATGACCTTT 211
Cfa    CCATTCTGTCACTTGCAGCGATAAAGCAGCTGAATGAGG-AGCAGCAGAAGATGGCCTTT 218
Eca    CCATTCTGTCACTTGCAGCGATAAAGCAGCTGAGTGAGGAGCCAGCAGAAGATGCCCTTT 222
Bta    CCATTCTGTCACTTGCAGTGATAAAGCAGCTGAGTGAGGAAGCAGCAGAAGATGCCCTTT 234
       ****************** ********     *  ***    ****** ** ** *****
```

```
Hsa    TAAAGTTATCA-TCCCGCAGGGCTTGTCACAGCCCCTCTCTGCCTTGGGCAGCCCCAACA 291
Ptr    TAAAGTTATCA-TCCCGCAGGGCTTGTCACAGCCCCTCTCTGCCTTGGGCAGCCCCAACA 291
Mma    TAAAGTTATCA-TCCCGCAGGGCTTGTCACAGCCCTTC--TGCCTTGGGCAGCCCCAACA 289
Cja    TAAAGTTATCA-TCCCACAGGGCTTGTCACAGCCCCTC--TGCCTCGGGCAGCCCCAACA 290
Bph    TAAAGTTATCAGTCCAGCC-AGCTCGCCACAGCCCC-T-CCGCCTTGAGCAGCTCCGGCA 281
Mmu    TAAAGTTATCAGTTCGGCAAAGCTTGTCACGGCCCCTT--TGCCTTGAGCAGCTCCACTC 284
Fca    TAAAGCTATCAGCCCAGCAGAGCCTGTCACTGCCCCCT-CCACCTTGAGCAGCCCCAACA 270
Cfa    TAAAGCTATCAGGCCCGCAGAGCCTGTCACTACCCC-T-CCACCTTGAGCAGCCC-AACA 275
Eca    TAAAGTTATCAGTCCAGCGGAACTTGTCACAGCCCC-T-CCGCCTTGAGCAGCCCCAACA 280
Bta    TAAAGTTATCAGTCCAGCC-TGCTTGTCACAGCCCC-T-CTGCCTTGAGCAGCCCCGACA 291
       ***** *****   *  *    *  * ***  ***       *** * ***** *

Hsa    GCCCCC------CAGACCCTCAGCTGCAAGAGCCGCATTCTTACTGATTTGACTCAAGAG 345
Ptr    GCCCCC------CAGACCCTCAGCTGCAAGAGCCGCATTCTTACTGATTTGACTCAAGAG 345
Mma    GCCCCC------CAGACCCTCGGCTGCAAGAGCCGCATTCTTGCTGATTTGACTCAAGAG 343
Cja    GCCCCC------CAGACCCTCAGCTGCAAGAGCTGAATTCTTGCTGATTTGACTCAAGAG 344
Bph    AGCCC-------GGGACCCTCAGCTGCAGGAGCCACATTCTTGTTGATTGGCCTCAAGAG 334
Mmu    ACCCCCCTCCCCCAGACCCTCAGCTGCAAGAGTCACATTCTTGCTGATTTGCCTCAAGAG 344
Fca    ACCCC---------AGACCCTCAGCTGCAAGAGCCACATTCTTGTTGATTTGCCTCAAGAG 322
Cfa    GCCCC---------GGACCCTCAGCGGCAAGAGCCACATTCTTGTTGATTTGCCTCAAGAG 327
Eca    ACCTCC------GGGACCCTCGGCTGCAAGAGCCACATTCTTGTTGATTTGCCTCAAGAG 334
Bta    ACCCCC------AGGACCCTCAGCTGCAGGAGCCACATTCTTGTTGATTTGCCTCAAGAG 345
         * *         ******* ** *** ***    ******  ***** * ********

Hsa    GCTCCCACTTCAAAGGTGTGA 366
Ptr    GCTCCCACTTCAAAGGTGTGA 366
Mma    GCTTCCACTTCAAAGGTGTGA 364
Cja    GCTCCCACTTCAAAGGTGTGG 365
Bph    GTTCCCACTTCAAAGGTGTGA 355
Mmu    GCCCCCACTTCAAAGGTGTGA 365
Fca    GTTCCCACTTCAAAGGTGTGA 343
Cfa    GTTCCCACTTCAAAGGTGTGA 348
Eca    GTTCCCACTTCAAAGGTGTGA 355
Bta    GTTCCCACTTCAAAGGTGTGA 366
       *   ******    *
```
